# Associations of lipoprotein subclasses with all-cause and cardiovascular mortality: results of two independent cohorts with a 20 year follow-up

**DOI:** 10.1186/s12944-025-02779-0

**Published:** 2025-11-14

**Authors:** Florian Fierfas, Martin Bahls, Ann-Kristin Henning, Astrid Petersmann, Kathrin Budde, Marcus Dörr, Henry Völzke, Matthias Nauck, Anke Hannemann, Nele Friedrich

**Affiliations:** 1 Institute of Clinical Chemistry and Laboratory Medicine, University Medicine Greifswald, Greifswald, Germany; 2 Department of Internal Medicine B - Cardiology, University Medicine Greifswald, Greifswald, Germany; 3 German Centre for Cardiovascular Research (DZHK), partner site Greifswald, Greifswald, Germany; 4 Institute for Clinical Chemistry and Laboratory Medicine, University Medicine Oldenburg, Oldenburg, Germany; 5 Institute for Community Medicine, University Medicine Greifswald, Greifswald, Germany

**Keywords:** Lipoprotein subclasses, Mortality, LDL triglycerides, Small/dense LDL particles

## Abstract

**Background:**

Currently total cholesterol (TC) and low-density lipoprotein cholesterol (LDL-C) are used in clinical practice to estimate future cardiovascular risk. We assessed whether other lipoprotein subclasses also contribute to cause-specific and all-cause mortality in the general population.

**Methods:**

Two independent cohorts of the Study of Health in Pomerania (SHIP-START and SHIP-TREND) were used. Participants were selected from population registration offices. The primary outcomes were all-cause, cardiovascular and cancer mortality. TC, total triglycerides (TG), phospholipids as well as the fractional concentrations of cholesterol, TG, phospholipids, and apolipoproteins of all lipoprotein subclasses were measured using nuclear magnetic resonance spectroscopy. Cox proportional hazard regression models were applied to assess the association between lipoprotein subclasses and mortality. Additionally, cause-specific hazards for cardiovascular disease (CVD) and cancer mortality were modelled considering competing events.

**Results:**

Data from 3,579 SHIP-START and 4,267 SHIP-TREND individuals were included. During follow-up, 946 (26.4%) SHIP-START and 387 (9.1%) SHIP-TREND participants died. In both cohorts, total LDL-TG and LDL1-TG to LDL6-TG but not total TG were positively or U-shaped related with all-cause mortality. In SHIP-START, total TG, VLDL-TG, IDL-TG and LDL-TG (including subclasses) were associated with CVD mortality. HDL4-C as well as small and dense LDL-C (e.g. LDL6-C) represented risk factors for mortality with mutually enhancing effects.

**Conclusions:**

The findings suggest that lipoprotein subclasses, especially LDL-TGs or HDL4-C/LDL6-C, provide information beyond the established TC and LDL-C levels and therefore might be of use for an early identification of subjects at risk.

**Supplementary Information:**

The online version contains supplementary material available at 10.1186/s12944-025-02779-0.

## Background

Lipoproteins are a heterogenous group of plasma molecules with crucial roles in lipid metabolism. Major particles include very low (VLDL), low (LDL) and high (HDL) density lipoproteins. Associations between HDL cholesterol (HDL-C) or LDL cholesterol (LDL-C) and cardiovascular diseases (CVD) or mortality are well known hence both are commonly used for individual cardiovascular risk estimation [1]. Generally HDL-C is seen as atheroprotective [2], whereas LDL-C is seen as atherogenic [1, 3]. This is not inherently appropriate, especially when chronic co-morbidities exist. In hemodialysis patients as well as in patients with non-diabetic chronic kidney disease (CKD), for example, the conventional lipid profile is insufficient to estimate mortality and CVD risk [4].

Classical lipoproteins can be further distinguished in specific subclasses according to their density and size e.g. LDL1 – LDL6. The Ludwigshafen Risk and Cardiovascular Health Study [5] based on more than 1,600 individuals, divided LDL particles in six subclasses and showed that small as well as large LDL particles were related to higher all-cause and CVD mortality. A recent NHANES III [6] investigation reported that very low and very high LDL-C levels were related to higher CVD mortality. These findings argue for a U-shaped association between LDL particle diameter as well as LDL-C and CVD mortality. Therefore, the assessment of lipoprotein subclasses may improve our understanding of lipid metabolism and to develop better risk estimates for CVD. A previous review [7] provided evidence for this presumption. The authors recommend a more thorough lipoprotein testing in case of family history of therapy resistance, unexplained premature coronary artery disease or rapid progression of atherosclerosis, as the additional information might result in treatment adaption. In another study [8], higher levels of small dense LDL were found to be more atherogenic and an absolute reduction of these subclasses via statins or other therapies were observed to be beneficial regarding CVD morbidity and mortality [9]. Next to particle size and density, also the composition of the LDL particles plays an important role. While LDL-C is an important cardiovascular risk factor, also the triglyceride (TG) content in LDL particles was shown to be related to atherosclerotic phenotypes [10]. With respect to HDL, two studies [11, 12] demonstrated abnormal HDL levels in CKD patients which were not atheroprotective, but related to impaired endothelial repair and function, increased systolic blood pressure, enhanced production of reactive oxygen species and inhibited nitric oxide bioavailability. In both investigations [11, 12], symmetric dimethylarginine (SDMA) was found as the responsible modulator. It is uncertain whether higher levels of SDMA may induce other sized HDL subclasses. Smaller HDL particles seem to be independent protective factors as they are inversely associated with time to adverse events in patients with heart failure with reduced (HFrEF) and preserved ejection fraction (HFpEF) [13] and low levels associate to an increased risk of long-term clinical events in secondary prevention [14]. However, there is also evidence that smaller HDL particles, along with large VLDL particles, are significantly associated with coronary calcification and development of occlusive disease [15, 16]. On the other hand, athero- and cardioprotective effects could be attributed to a greater extent to larger HDL [17].

Taken together, the current evidence on the associations of lipoprotein subclasses with mortality is inconsistent. Therefore, the aim of the present study was to analyze the associations between lipoprotein subclasses and all-cause mortality, CVD and cancer mortality in two independent cohorts.

## Methods

### Study population

Data were obtained from two population-based cohort studies: the Study of Health in Pomerania (SHIP-START) and SHIP-TREND. SHIP-START and SHIP-TREND data were collected in West Pomerania, a region in the northeast of Germany [18]. Both studies complied with the Declaration of Helsinki and were approved by the ethics committee of the University of Greifswald. All participants provided written informed consent. A detailed description is given in the supplement.

Of the 4,308 SHIP-START participants, 372 individuals without ^1^H Nuclear magnetic resonance (NMR) measurements and additionally 357 individuals with missing data for confounding factors were excluded. Thus, the final study population for the present analyses consisted of 3,579 individuals. Of the 4,420 SHIP-TREND participants, 80 individuals without NMR measurements and additionally 73 individuals with missing data for confounding factors were excluded. Thus, the final study population for the present analyses consisted of 4,267 individuals.

### Measurements

Information on age, sex, socio-demographic characteristics, lifestyle parameters and medical histories were assessed by computer-aided personal interviews. Smoking status was assessed by self-report (current, former, and never-smokers). Mean daily alcohol consumption was calculated using beverage-specific pure ethanol volume proportions. Individuals who did not participate in physical exercise during summer and winter for at least 1 h/week were classified as being physically inactive. Waist circumference was measured to the nearest 0.1 cm using an inelastic tape, midway between the lower rib margin and the iliac crest in the horizontal plane. After a 5-minute resting period, systolic and diastolic blood pressure (BP) was measured three times on the right arm of seated individuals using a digital BP monitor (HEM-705CP, Omron Corporation, Tokyo, Japan) with each reading being followed by a further resting period of three minutes. The mean of the second and third measurement were used for analyses. The definition of diabetes mellitus was based on a self-reported physician’s diagnosis, glycated hemoglobin (HbA1c) ≥ 6.5%, glucose ≥ 11.1mmol/l or self-reported use of antidiabetic medication in the last 7 days. Renal diseases were defined as self-reported renal diseases or estimated glomerular filtration rate < 60 ml/min/1.73 m² (MDRD equation). The definition of liver diseases was based on self-reported liver cirrhosis or atrophy of the liver. Additionally, all individuals with serum gamma-glutamyl transferase, aspartate-amino transferase, or alanine-amino transferase levels > population mean + 2*standard deviations (SD) were classified as individuals with liver diseases. CVD was defined as self-reported history of myocardial infarction, stroke, or angina pectoris as well as the presence of a cardiac pacemaker or N-terminal prohormone of brain natriuretic peptide levels ≥ 125pg/ml.

### Follow-up of vital status

Information on the participant’s vital status was acquired at annual intervals from the time of enrolment through January, 2023. Individuals were censored at either death or failure to follow-up. The number of months between the baseline examination and censoring was used as follow-up length. Death certificates were requested from the local health authority of the residence of death. The underlying causes of death were independently defined by two internists. In case of disagreement, a joint reading was performed. Causes of death were categorized according to the International Classification of Diseases, 10th revision (ICD10). CVD included codes I10 to I79 and R96 and cancer C00 to C97.

### Lipoprotein subclasses

^1^H NMR spectra were recorded on a Bruker AVANCE-II 600 NMR spectrometer operated by TOPSPIN 3.2 software (both Bruker Biospin, Rheinstetten, Germany), equipped with 5-mm z-gradient probe (Bruker Biospin, Rheinstetten, Germany) and an automated tuning and matching (ATMA) unit. In cooperation with the Institute of Clinical Chemistry and Laboratory Medicine at the University Medicine Greifswald, Bruker developed an automatic analysis tool to quantify lipoprotein subclasses from NMR spectra. In the framework of this process the lipoprotein measurements were performed by ultracentrifugation, the gold standard method to analyse lipoproteins, in Greifswald. Based on this development, the spectra of the present study were submitted to data analysis for lipoprotein subclass analysis B.I.LISA^™^ (Bruker BioSpin GmbH, Rheinstetten, Germany). A detailed description is given in the supplement.

### Statistical analysis

Categorical data were expressed as percentages; continuous data were expressed as median (Q1; Q3). For bivariate analyses the Mann-Whitney test (continuous data) or Chi-Quadrat-test (nominal data) were used. Multivariable Cox proportional hazard regression models with event time as censoring variable were run to assess the associations between lipoprotein subclasses and all-cause mortality. For CVD and cancer mortality, we calculated cause-specific hazard models. Cause-specific hazards were modelled for each event separately and thus, the respective competing events were considered as censored cases. The selection of the covariables was performed based on a directed acyclic graph (DAG) (figure S1). The models were adjusted for sex, age, waist circumference, smoking, physical inactivity, alcohol consumption, diabetes mellitus, renal diseases, liver diseases, CVD and systolic BP. In addition to examining linear associations, we employed restricted cubic splines with three knots to explore potential non-linear relationships between confounders, lipoprotein subclasses, and mortality. The analysis followed a stepwise procedure. In the first step, each confounder was individually evaluated by fitting both a linear and a spline model, which were then compared using a likelihood ratio test (LRT). Based on the result of the LRT the confounders were either used as linear or spline term. In a second step, in the adjusted model, the linear and spline models for the lipoprotein subclasses were subsequently compared using LRTs. To prevent overfitting, we additionally conducted a visual inspection of the model fits. Table S1 presents the used models for all analyses. In an additional analyses based on the Cox regression results, Kaplan-Meier survival curves were used to illustrate the association between the combination of HDL4-C/LDL6-C and all-cause as well as CVD mortality in SHIP-START. Finally, the effects of a one SD increase in the log(HDL4-C/LDL6-C) ratio on all-cause and CVD mortality was examined in SHIP-START using adjusted Cox regression models. Hazard ratios (HR) with 95% confidence intervals (CI) were calculated. To account for multiple testing, we adjusted the P values from the regression analyses by controlling the false discovery rate (FDR) at 5% using the Benjamini-Hochberg procedure. Statistical analyses were performed with SAS 9.4 (SAS Institute Inc., Cary, NC, USA).

## Results

### General characteristics and follow-up of vital status

Baseline characteristics for both study populations as well as for living and deceased individuals are provided in Table 1. SHIP-TREND participants were, on average, two years older than SHIP-START participants, whereas the sex-distribution was similar between the cohorts. Furthermore, SHIP-TREND participants were less often current smokers, less physically inactive, drank less alcohol, had a slightly higher average waist circumference and had lower mean systolic blood pressure compared to SHIP-START participants. With respect to comorbidities, SHIP-TREND individuals were more often affected by diabetes mellitus and liver diseases but less often by renal diseases.

**Table 1:** Baseline characteristics of the study populations and vital status

	SHIP-START (*n* = 3,579)	SHIP-TREND (*n* = 4,267)	SHIP-START			SHIP-TREND		
			alive (*n* = 2,633)	deceased (*n* = 946)	p*	alive (*n* = 3,880)	deceased (*n* = 387)	p*
Age, years	50 (36; 63)	52 (40; 64)	42 (32; 55)	67 (60; 74)	< 0.01	51 (38; 62)	70 (62; 77)	< 0.01
Men, % (n)	49.0 (1,755)	48.5 (2,071)	44.6 (1,173)	61.5 (582)	< 0.01	46.7 (1,811)	67.2 (260)	< 0.01
Smoking, % (n)					< 0.01			< 0.01
Never smoker	36.0 (1,286)	36.5 (1,557)	36.7 (966)	33.8 (320)		37.2 (1,443)	29.5 (114)	
Ex-smoker	33.3 (1,193)	36.7 (1,564)	29.8 (785)	43.1 (408)		35.7 (1,385)	46.3 (179)	
Current smoker	30.7 (1,100)	26.9 (1,146)	33.5 (882)	23.0 (218)		27.1 (1,052)	24.3 (94)	
Physical activity, % (n)	42.8 (1,533)	69.3 (2,956)	47.7 (1,255)	29.4 (278)	< 0.01	69.6 (2,699)	66.4 (257)	0.20
Alcohol consumption, g/day	5.4 (1.3; 15.0)	3.4 (0.7; 10.6)	6.5 (2.0; 16.0)	3.2 (0; 11.8)	< 0.01	3.4 (0.7; 10.6)	2.7 (0; 10.9)	0.06
Waist circumference, cm	89 (79; 99)	91 (80; 101)	86 (76; 96)	96 (87; 105)	< 0.01	90 (79; 100)	99 (90; 108)	< 0.01
Systolic BP, mmHg	135 (121; 149)	127 (115; 140)	130 (118; 144)	146 (132; 159)	< 0.01	127 (114; 139)	135 (123; 148)	< 0.01
Diabetes mellitus, % (n)	10.5 (375)	12.4 (530)	5.1 (133)	25.6 (242)	< 0.01	10.4 (403)	32.8 (127)	< 0.01
Liver diseases, % (n)	5.9 (210)	6.4 (503)	5.1 (134)	8.0 (76)	< 0.01	11.2 (434)	17.8 (69)	< 0.01
Renal diseases, % (n)	17.0 (609)	12.8 (545)	12.0 (315)	31.1 (294)	< 0.01	10.5 (407)	35.7 (138)	< 0.01
Lipid-lowering medication, % (n)	8.6 (309)	14.1 (601)	5.3 (139)	18.0 (170)	< 0.01	11.9 (460)	36.4 (141)	< 0.01
CVD, % (n)	23.6 (844)	30.0 (1,248)	14.5 (382)	48.8 (462)	< 0.01	26.6 (1,032)	63.6 (246)	< 0.01
*Mortality*								
Follow-up time, years	20.2 (18.7; 21.9)	9.0 (7.5; 11.7)	-	-		-	-	
All-cause mortality, % (n)	26.4 (946)	9.1 (387)	-	-		-	-	
CVD mortality, % (n)	8.8 (310)	2.2 (91)	-	-		-	-	
Cancer mortality, % (n)	8.6 (303)	2.4 (100)	-	-		-	-	
^*1*^ *H-NMR-based lipids*								
Total cholesterol, mg/dl	221 (192; 251)	216 (185; 250)	218 (189; 249)	227 (199; 258)	< 0.01	217 (186; 251)	204 (171; 240)	< 0.01
LDL cholesterol, mg/dl	115 (94; 136)	129 (105; 155)	114 (93; 135)	119 (98; 142)	< 0.01	130 (106; 156)	119 (94; 149)	< 0.01
HDL cholesterol, mg/dl	56 (48; 66)	58 (49; 70)	57 (49; 67)	52 (45; 62)	< 0.01	59 (50; 70)	54 (45; 62)	< 0.01
Triglycerides, mg/dl	128 (93; 182)	113 (79; 163)	122 (89; 175)	143 (109; 202)	< 0.01	111 (78; 161)	130 (98; 186)	< 0.01

Continuous data are expressed as median (Q1; Q3); nominal data are given as percentages (absolute number). *Chi-Squared test (nominal data) or Mann-Whitney test (interval data) were performed. BMI = body mass index, BP = blood pressure, CVD = cardiovascular disease, ^1^H-NMR = nuclear magnetic resonance, LDL = low density lipoprotein, HDL = high density lipoprotein.

A major difference between the cohorts was found for the proportion of individuals taking lipid-lowering medication with a much higher proportion in SHIP-TREND. Regarding lipid levels, SHIP-TREND participants showed higher LDL-C and HDL-C levels as well as slightly lower total cholesterol and lower TG levels compared to SHIP-START participants. In both study populations, deceased individuals exhibited less favourable profiles across nearly all health parameters compared to surviving individuals.

In SHIP-START during the 67,066 person-years of follow-up 946 (26.4%) individuals out of 3,579 participants died. CVD and cancer caused 310 (32.7%) and 303 (32.0%) deaths, respectively. In SHIP-TREND, the follow-up was on average 9 years shorter. Therefore, during the 39,576 person-years of follow-up 387 (9.1%) individuals out of 4,267 participants died. CVD and cancer caused 91 (23.5%) and 100 (25.8%) deaths, respectively.

### All-cause mortality

**Fig. 1:**
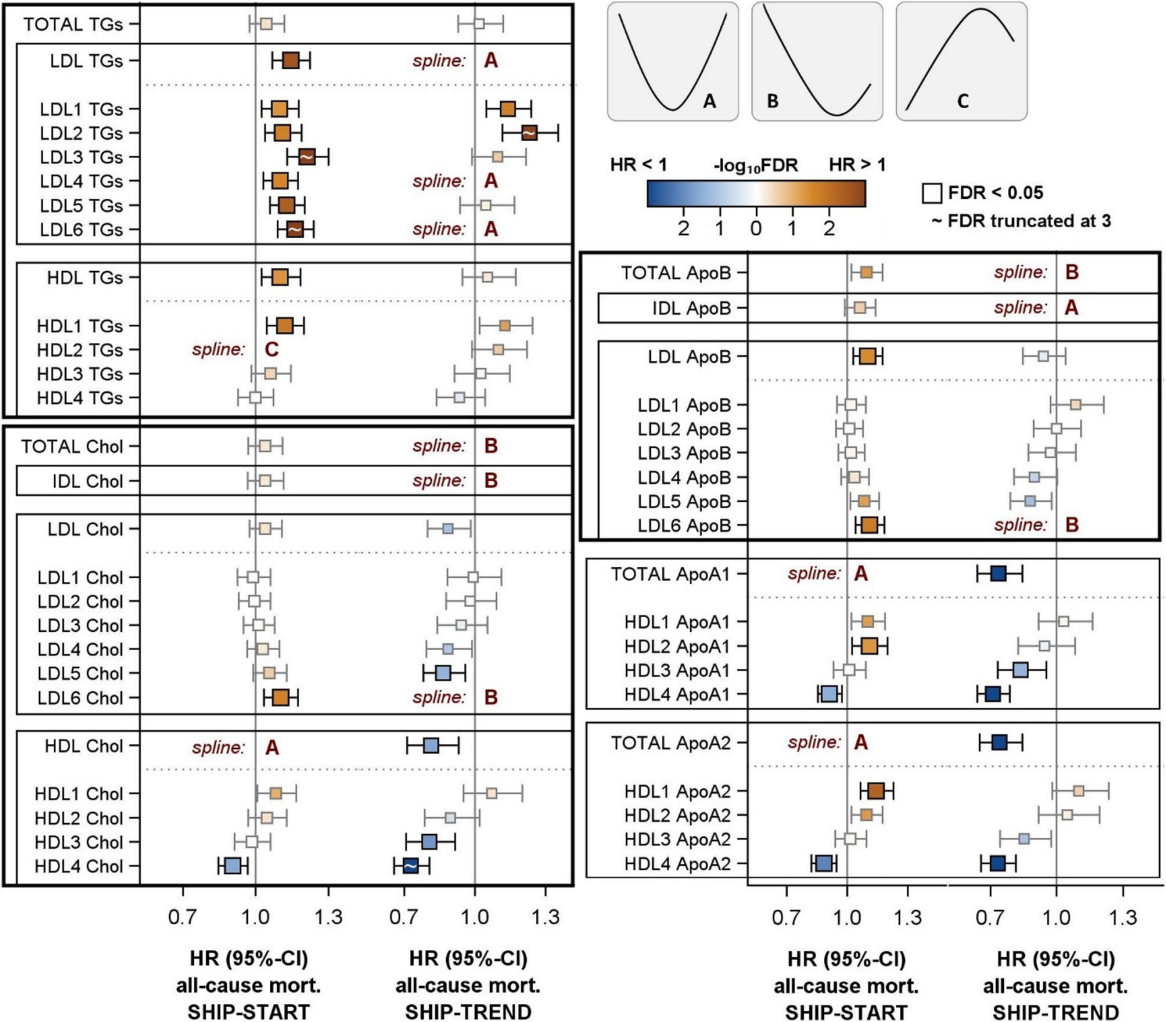
Cox regression models for the associations of selected lipoprotein subclasses with all-cause mortality in SHIP-START and SHIP-TREND. Hazard ratios (HR) with 95% confidence intervals (CI) for one standard deviation increase in lipoprotein subclasses are displayed. Models were adjusted for age, sex, waist circumference, smoking, physical activity, alcohol consumption, diabetes mellitus, renal diseases, liver diseases, cardiovascular disease and systolic blood pressure. When appropriate, spline terms for age, waist circumference and alcohol consumption were included. Models with a spline term for lipoproteins (see method section) are marked by spline and function form (see upper right part of the figure). *TGs *= triglycerides, *Chol* = cholesterol, *IDL* = intermediate density lipoprotein,* LDL* = low density lipoprotein, *HDL* = high density lipoprotein. The illustration was limited to lipoprotein subclasses that demonstrated statistically significant relations with all-cause mortality. Full results are displayed in Figure S2.

**Fig. 2:**
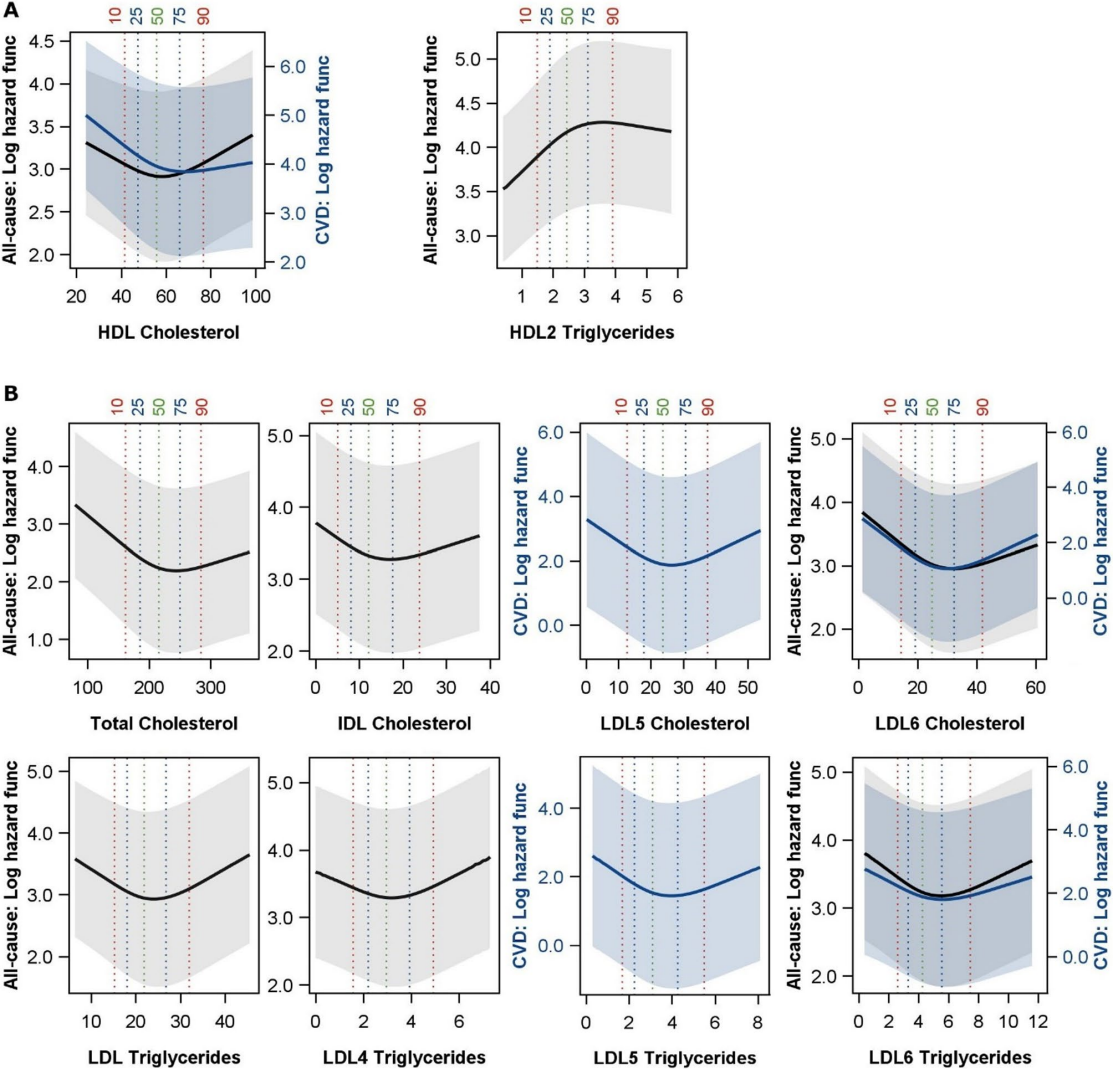
Predicated log hazard function for all-cause (black) and/or cardiovascular disease (CVD) mortality (blue) with 95% confidence interval (shaded area) as a function of the lipoprotein subclasses in SHIP-START (A) and SHIP-TREND (B) for selected non-linear associations (see Figs. 1 and 2). 10%, 25%, median, 75% and 90% percentile are displayed. The displayed range of the lipoprotein subclasses was restricted to the mean ± 3 standard deviations for simplification. Cox regression models were adjusted for age, sex, waist circumference, smoking, physical activity, alcohol consumption, diabetes mellitus, renal diseases, liver diseases, cardiovascular disease and systolic blood pressure. When appropriate, spline terms for age, waist circumference and alcohol consumption were included. Apolipoproteins are displayed in Figure S4. *IDL* = intermediate density lipoprotein, *LDL* = low density lipoprotein, *HDL* = high density lipoprotein

Results of multivariable Cox models for all-cause mortality are presented in Figs. 1 and 2, supplementary figures S2 and S4 as well as in supplementary tables S1 und S2. In SHIP-START, after adjustment for relevant confounders, LDL-TG and the TG content in all LDL subclasses were positively associated with all-cause mortality (Fig. 1). In addition, HDL-TG and HDL1-TG exhibited positive and HDL2-TG an inverted reverse J-shaped association with all-cause mortality (Fig. 2). The relations to total TG, VLDL-TG and IDL-TG missed statistical significance (figure S2). Similar findings became apparent in SHIP-TREND for TG content in LDL1 and LDL2 subclasses. However, LDL-TG as well as LDL4-TG and LDL6-TG showed U-shaped relations with all-cause mortality.

With respect to cholesterol, we observed different results between the study populations. In SHIP-START no relations of total cholesterol, VLDL-C, IDL-C and LDL-C with all-cause mortality were observed. In SHIP-TREND total cholesterol and IDL-C showed reverse J-shaped associations. Furthermore, the cholesterol content in LDL subclasses showed different association patterns with a positive association for LDL6-C in SHIP-START and inverse or reverse J-shaped relations between LDL5-C or LDL6-C and all-cause mortality in SHIP-TREND.

In SHIP-START, HDL-C exhibited a U-shaped association with all-cause mortality with the lowest risk around 60 mg/dl (Fig. 2), while HDL4-C was inversely linked to all-cause mortality (Fig. 1). In SHIP-TREND, the inverse association for HDL4-C was confirmed and further inverse relations were seen for HDL3-C and HDL-C. Analyses for Apo-B, representing the LDL particle number, yielded similar results to the cholesterol content in LDL particles (Fig. 1 and S2). In SHIP-START, higher numbers of LDL particles as well as small and dense LDL6 particles were positively related to all-cause mortality. SHIP-TREND the number of LDL6 particles was U-shaped linked to all-cause mortality. Furthermore, also total Apo-B and IDL Apo-B showed U-shaped and reverse J-shaped associations, respectively. Analyses for total Apo-A1 and Apo-A2 as well as HDL3/4 Apo-A2 and Apo-A2 support the findings for HDL-C being a protective marker regarding all-cause mortality by finding inverse associations in SHIP-TREND. In SHIP-START, the inverse link to HDL4 Apo-A1 and Apo-2 were confirmed. Furthermore, positive associations between HDL2 Apo-A1 and HDL1 Apo-2 with all-cause mortality became apparent. These findings result in overall U-shaped relations of total Apo-A1 and Apo-A2 with mortality (figure S4).

### CVD mortality

**Fig. 3:**
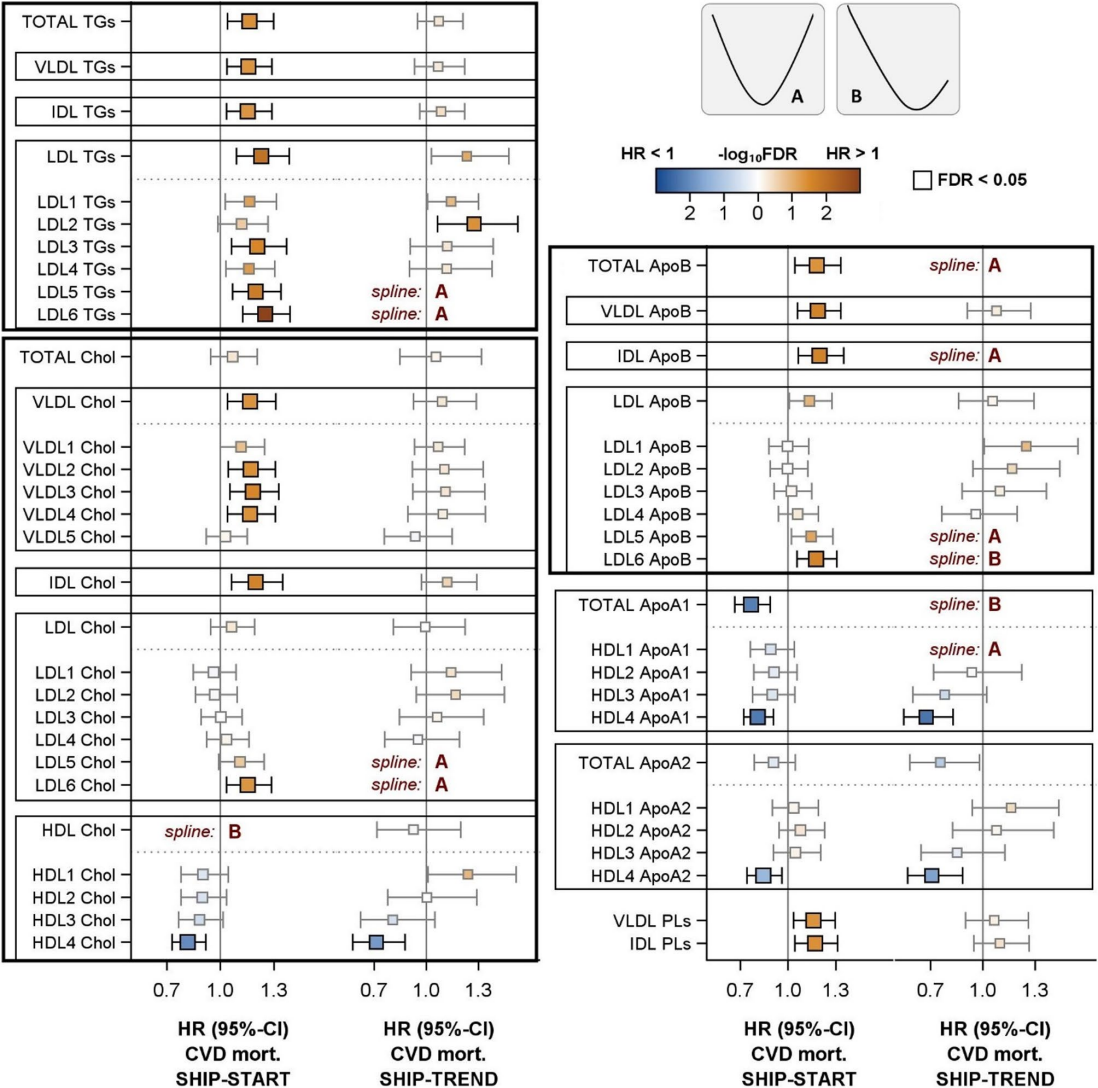
Cox regression models for the associations of selected lipoprotein subclasses with CVD mortality in SHIP-START and SHIP-TREND. Hazard ratios (HR) with 95% confidence intervals (CI) for one standard deviation increase in lipoprotein subclasses are displayed. Models were adjusted for age, sex, waist circumference, smoking, physical activity, alcohol consumption, diabetes mellitus, renal diseases, liver diseases, CVD and systolic blood pressure. When appropriate, spline terms for age, waist circumference and alcohol consumption were included. Models with a spline term for lipoproteins (see method section) are marked by spline and function form (see upper right part of the figure). *CVD* = cardiovascular disease, *TGs* = triglycerides, *Chol* = cholesterol, *VLDL* = very low density lipoprotein, *IDL* = intermediate density lipoprotein, *LDL* = low density lipoprotein, *HDL* = high density lipoprotein, *PL* = Phospholipids. The illustration was limited to lipoprotein subclasses that demonstrated statistically significant relations with CVD mortality. Full results are displayed in Figure S3

Results of multivariable Cox models for CVD mortality are presented in Figs. 2 and 3 and S3 as well as in supplementary tables S1 and S2. In SHIP-START, total TGs and the TG content in VLDL, IDL and LDL including subclasses were positively linked to CVD mortality. Moreover, VLDL-C, VLDL2-C to VLDL4-C, IDL-C and LDL6-C but not total cholesterol and LDL-C were positively related with CVD mortality. Similar relations were seen for the LDL6 particle number, i.e. the LDL6 Apo-B content. Furthermore, total Apo-B, VLDL Apo-B and IDL Apo-B were linked to CVD mortality in SHIP-START. In contrast, in SHIP-TREND U-shaped associations to CVD mortality became apparent for LDL-5 and LDL-6 particle number, TG and cholesterol content as well as total and IDL Apo-B.

With respect to HDL particles, HDL4-C were inversely associated with CVD mortality in both study populations. A comparable association pattern was observed for the Apo-A1/Apo-A2 content in the HDL4 subclass. Furthermore, U-shaped or reverse J-shaped relations of total HDL-C and Apo-A1 as well as HDL1 Apo-A1 with CVD mortality were observed in at least one of the study populations.

In contrast to all-cause mortality, the VLDL and IDL phospholipid content was positively linked to CVD mortality in SHIP-START but not in SHIP-TREND (Fig. 3).

### Cancer mortality

Results of multivariable Cox models for cancer mortality are presented in figure S5 as well as in supplementary table S1. Both studies revealed only a few non-consistent significant findings. In SHIP-TREND, for example, total HDL-C as well as HDL3-C and HDL4-C were inversely related to cancer mortality after correction of multiple testing. None of these associations was reproduced in.

SHIP-START. Further, in SHIP-TREND but not in SHIP-START, total Apo-A1 and total Apo-A2 were inversely related to cancer mortality but none of the subclasses demonstrated a similar association.

### Combination of LDL6-C and HDL4-C

**Fig. 4:**
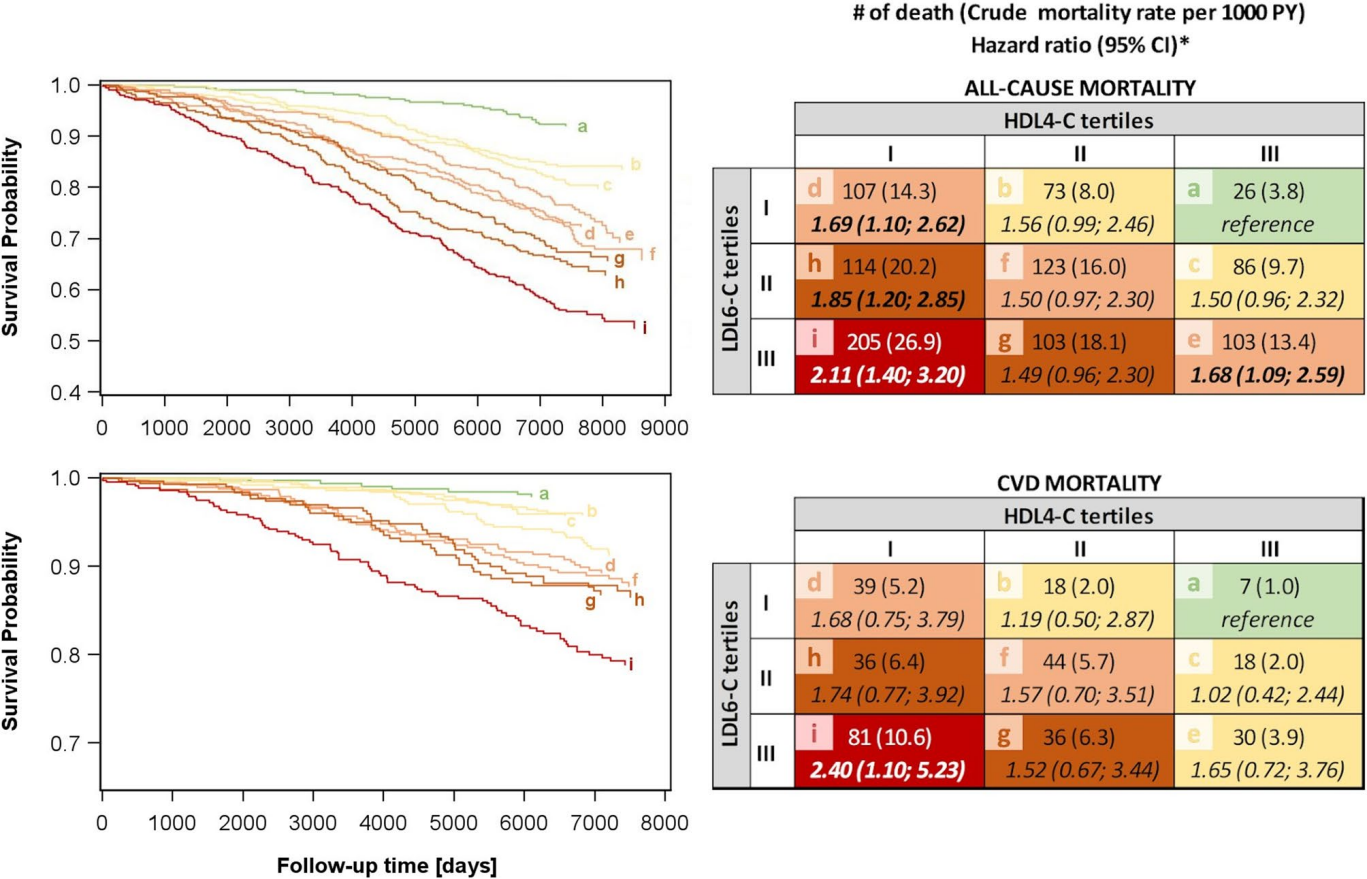
Left side: Kaplan-Meier survival curves for all-cause (upper row) and CVD (lower row) mortality in SHIP-START. Right side: Number of death (crude mortality rates) and hazard ratio with 95% confidence interval (CI) for all-cause (upper row) and CVD (lower row) mortality. Both, curves and rates, were displayed for the combined consideration of the cholesterol content in LDL6 (LDL6-C) and HDL4 (HDL4-C) particles in SHIP-START. *LDL* = low density lipoprotein, *HDL* = high density lipoprotein, *PY* = person years. *Cox regression models were adjusted for age, sex, waist circumference, smoking, physical activity, alcohol consumption (linear and spline term), diabetes mellitus, renal diseases, liver diseases, cardiovascular disease and systolic blood pressure

In our analyses, the cholesterol content in LDL6 as well as HDL4 represented risk markers for all-cause and CVD mortality. The combined effect of these risk markers was examined in SHIP-START. Here, we found, that individuals with high LDL6-C and simultaneously low HDL4-C had by far the highest all-cause and CVD mortality compared to other LDL6-C/HDL4-C combinations (Fig. 4). In turn, the negative effect of either high LDL6-C or low HDL4-C is attenuated by lower LDL6-C or higher HDL4-C levels, respectively. The crude mortality rates, per 1,000 person-years, were lowest among individuals with low LDL6-C/high HDL4-C (all-cause mortality: 3.8, CVD mortality: 1.0), intermediate in individuals with a high LDL6-C/high HDL4-C (all-cause mortality: 13.4; CVD mortality: 3.9) and highest in individuals with high LDL6-C/low HDL4-C (all-cause mortality: 26.9, CVD mortality: 10.6).

These findings were supported by the results obtained from adjusted Cox regression models (Fig. 4). Subjects with high LDL6-C/low HDL4-C had the highest risk for all-cause [HR 2.11 (95%-CI 1.40–3.20)] and CVD [HR 2.40 (95%-CI 1.10–5.23)] mortality compared to subjects in the reference category (lowest LDL6-C/highest HDL4-C). Subjects with high LDL6-C/low HDL4-C further had the highest median log(HDL4-C/LDL6-C) ratio compared to all other groups (figure S6). Finally, a one SD increase in the log(HDL4-C/LDL6-C) ratio was related to an adjusted HR of 1.11 (95%-CI 1.05–1.18) and 1.17 (95%-CI 1.06–1.29) for all-cause and CVD mortality, respectively.

## Discussion

The present study investigated the relation of lipoprotein subclasses and their content of TG, cholesterol and apolipoproteins with all-cause and cause-specific mortality in two independent cohorts. Due to recent findings promoting the idea of U- or inverse U-shaped risk profiles of lipids in relation to CVD or mortality [6, 19–22] we put a focus on possible non-linear associations.

### Standard lipid parameters and mortality

The standard clinical parameters to examine an individual’s lipoprotein profile are total TGs as well as total cholesterol, LDL-C and HDL-C. Our observed positive association of total TGs with CVD mortality is in agreement with previous findings [23–26], while an association with all-cause mortality was absent. Associations between total cholesterol or LDL-C with all-cause or CVD mortality were also absent. This was surprising given that a reduction of both via statins significantly reduces all-cause and CVD mortality especially in patients with higher LDL-C [3, 25, 27–29].

Our finding that total HDL-C exhibits an inverse association with CVD mortality, that reaches a plateau at high concentrations, is in line with the current consensus [2, 24, 25, 29, 30]. The U-shaped association to all-cause mortality in SHIP-START and the inverse association in SHIP-TREND point to the fact that low HDL-C is related to increased all-cause mortality. The direction of the effect in higher HDL-C is, however, inconsistent in our study. Previous studies [20–22, 31–34], reported U-shaped associations to all-cause mortality, similar to SHIP-START, but the underlying pathophysiology remains incompletely understood. An explanatory approach from an experimental study [35] states that high HDL-C impairs endothelial progenitor cells and angiogenesis, which in turn, supports the absence of protective effects of pharmacological elevation of HDL-C on mortality [29, 36]. Other mechanisms explaining the association of high HDL-C and CVD, include genetic variants, e.g. CETP, ABCA1, LIPC, SCARB1, that lead to extremely high HDL-C concentrations and a higher risk of coronary artery disease and death [21]. Moreover, with very high HDL-C levels, lipoproteins may be less active or more dysfunctional which might be related to a disturbed lipid metabolism.

### Lipoprotein/Apolipoprotein subclass profile and mortality

#### Triglyceride content

The negative properties of TGs regarding all-cause mortality seem to be mediated by the LDL-TG content. The LDL-TG subclasses showed mainly strong positive associations with all-cause mortality, even after adjustment for potential confounders. Similar associations were found for CVD mortality, while total TGs were only associated with CVD mortality in one cohort. Our findings are in line with results obtained in a large Danish study showing a link between LDL-TG and an increased atherosclerotic risk [37]. As discussed by the authors, an insufficient lipoprotein lipase activity, e.g. in obesity or CKD, might result in elevated LDL-TG levels and the increased atherosclerotic risk. LDL-TG could therefore be a surrogate marker for TG-rich remnant lipoproteins or alternatively favor local TG degradation and formation of plaques [37]. As to why significance for total TG to all-cause mortality was not present remains unclear. It can, however, be speculated that the effects of non-CVD mortality or the effects of adjusting for severe chronic diseases, contributed to a decreased overall impact of higher total TG.

#### Cholesterol content

In SHIP-START and SHIP-TREND, the total cholesterol content in LDL particles was neither related to all-cause nor to CVD mortality. With respect to the cholesterol content in LDL subclasses, the study populations revealed slightly different results. Higher LDL6-C was, for example, linked to a higher all-cause/CVD mortality in SHIP-START but showed a reverse J-shaped (all-cause) or U-shaped (CVD mortality) association in SHIP-TREND. In both populations, the lowest risks were found for LDL6-C levels between the median and 3rd quartile. These findings confirmed the results of a previous large prospective cohort study with respect to total LDL-C [38]. The authors reported that the lowest all-cause mortality risk was found at medium levels of around 140–143 mg/dL total LDL-C, which is twice as high as the recommended therapy level in very high-risk cases in secondary CVD prevention [39]. The association between low LDL-C levels and a higher mortality risk might be explained by reverse causation, as severe and chronic diseases impact on cholesterol parameters [40, 41]. The strong positive associations of LDL6-C in SHIP-START are in line with the growing body of evidence that elevated levels of smaller LDL particles are accompanied by an increased (CVD) mortality [9, 42–46]. Our finding of a positive linear association between LDL6 Apo-B and all-cause as well as CVD mortality further supports this. One LDL particle (also VLDL and IDL) has exactly one Apo-B molecule, which is consequently a marker for the particle number. The associations of Apo-B content in the LDL subclasses mainly mimic the results found for their cholesterol content. Not surprisingly, the LDL particle number is associated with a higher risk of CVD mortality [20, 29, 47].

LDL6 is the smallest LDL particle and has a lower cholesterol transport capacity per particle, therefore it is likely that higher LDL6-C results from an increasing LDL6 particle number. This assumption was confirmed in the present study (figure S7). Especially in SHIP-START, study participants who died in the observation period had higher baseline total LDL-C levels due to a higher LDL particle number (Apo-B level). This was particularly evident for LDL6 particles and LDL6-C levels. Interestingly, these differences were less strong in SHIP-TREND, which might explain the discrepancy regarding the association between LDL-C content and mortality in the two study populations. The role of small dense LDL particles in CVD, especially in atherosclerosis and coronary heart disease, is well discussed [48]. For example, small dense LDL particles are linked to enhanced arterial wall penetration, to oxidative modifications and exhibit a reduced affinity for the LDL receptor leading to a prolonged plasma retention [42].

With respect to HDL subclasses, strong inverse associations became apparent for HDL4-C with CVD mortality in both study populations. These results are in line with other findings depicting small dense HDL-C to be a better predicator for survival [13, 49–51] and reduced levels to correlate with an unfavorable outcome [14, 52–54]. For all-cause mortality, total HDL-C showed inverse (SHIP-TREND) or U-shaped (SHIP-START) associations, while HDL4-C was inversely associated in both study populations. The reason for the seemingly unfavorable effects of higher total HDL-C levels in SHIP-START on all-cause mortality is unclear. However, not examined non-CVD deaths might represent a plausible underlying cause.

Apo-A1 and Apo-A2 are the most dominant apolipoproteins of HDL particles and therefore markers for the particle number, even if they are not necessarily found in a one-to-one ratio like Apo-B in non-HDL particles. Both Apo-A1 and Apo-A2 are essential for HDL metabolism. The demonstrated inverse (SHIP-START) or reversed J-shaped (SHIP-TREND) associations between total Apo-A1, i.e. HDL particle number, and CVD mortality is coherent with recent studies [13, 20, 49]. Hence, the analyses of the Apo-A1 and Apo-A2 content support our hypothesis, that more dense HDL particles are linked to a lower mortality risk, whereas less dense HDL particles do not show any or might even show adverse effects. This might also explain the detected U-shaped relations as typical extremely high HDL-C values are accompanied by highly elevated levels of large HDL particles which have less capacity to acquire free cholesterol and efflux cholesterol [19].

Large parts of our findings are in line with a recent prospective study on individuals with type 2 diabetes [55]. That study also found denser HDL particles to be inversely associated to mortality, while larger HDL particles were positively associated. We replicated the seemingly unfavorable properties of larger HDL for all-cause mortality by measuring Apo-A2 content in SHIP-START and for CVD mortality for HDL1-C in SHIP-TREND, although the latter association barely missed statistical significance.

LDL as well as IDL and VLDL particles carry hepatic cholesterol anterograde to the peripheral tissue for various functions whereas HDL particles carry excess retrograde. A dysregulated cholesterol transport is involved in the development of atherosclerosis. Smaller denser LDL infiltrate easier and remain longer in the vascular endothelium and subendothelial space. Reduced clearance by the LDL receptor and higher vulnerability to oxidation make them more likely to be scavenged by macrophages [56] and more susceptible to denaturation by free radicals, thus promoting atherosclerosis. Therefore, HDL4 may better fetch excess cholesterol on time. In light of our strong findings for LDL6-C and HDL4-C, we found the combination of both to be more valuable than the individual components (Fig. 4, S6). The combination of high LDL6-C and low HDL4-C is related to even higher hazard rates compared to high LDL6-C and low HDL4-C alone.

### Strengths and limitations

The major strength of our study is the analysis of two large independent cohorts, which enabled us to replicate our results. Furthermore, both study populations were characterized by a long follow-up (up to 20 years) and a comprehensive lipidprotein subclass profile measured by ^1^H-NMR spectroscopy. The shorter follow-up in SHIP-TREND might, however, have been responsible for the slightly different results between the cohorts. A DAG was used to select the used confounders in the performed Cox regression analyses. DAGs provide a clear and structured theoretical framework for identifying confounders and determining appropriate adjustment sets, thereby strengthening causal inference. Their main advantage lies in making assumptions explicit and enhancing transparency and reproducibility. However, DAGs depend heavily on the correctness of the assumed structure and too many variables risks overfitting. Based on the DAG, we adjusted for a broad range of confounding factors. Yet, we cannot exclude residual confounding. Further, unmeasured or unknown factors might partially or even fully explain discrepant results. Another limitation arises from the fact, that we could not discriminate the cause of death beyond CVD and cancer.

### Conclusion

Our findings suggest that lipid subclassification provides information beyond established lipid parameters. Particularly LDL-TG regarding all-cause mortality, as well as LDL6-C/HDL4-C regarding all-cause and CVD mortality are promising markers to improve CVD risk estimation algorithms and improve early identification of patients at risk.

## Supplementary Information

Supplementary Material 1

## Data Availability

Restrictions apply to the availability of data generated or analyzed during this study to preserve patient confidentiality or because they were used under license. Data can be applied for following a standardized procedure: http://www2.medizin.uni-greifswald.de/cm/fv/ship/daten-beantragen/.
