# Supplementary material for: Associations of lipoprotein subclasses with all-cause and cardiovascular mortality: results of two independent cohorts with a 20 year follow-up
Source: Lipids Health Dis. 2025 Nov 14;24:363. doi: 10.1186/s12944-025-02779-0 (PMC12619359; doi:10.1186/s12944-025-02779-0)
Supplement: Supplementary file 1 — Supplementary Material 1 [file 12944_2025_2779_MOESM1_ESM.pdf]

SUPPLEMENT

Associations of lipoprotein subclasses with all-cause and cardiovascular mortality:

Results of two independent cohorts with a 20 year follow-up

Florian Fierfas[a], Martin Bahls[b,c], Ann-Kristin Henning[a], Astrid Petersmann[a,d], Kathrin Budde[a], Marcus Dörr[c, e], Henry Völzke[e], Matthias Nauck[a,c], Anke Hannemann[a,c], Nele Friedrich[a,c]

a - Institute of Clinical Chemistry and Laboratory Medicine, University Medicine Greifswald, Greifswald, Germany
b - Department of Internal Medicine B - Cardiology, University Medicine Greifswald, Greifswald, Germany
c - German Centre for Cardiovascular Research (DZHK), partner site Greifswald, Greifswald, Germany
d - Institute for Clinical Chemistry and Laboratory Medicine, University Medicine Oldenburg, Germany
e - Institute for Community Medicine, University Medicine Greifswald, Greifswald, Germany

METHODS

Study population

Data were obtained from two population-based cohort studies: the Study of Health in Pomerania (SHIP-START) and SHIP-TREND. SHIP-START and SHIP-TREND data were collected in West Pomerania, a region in the northeast of Germany [1, 2]. Samples were selected from the population registration offices (SHIP-START: two-stage stratified cluster sample; SHIP-TREND: stratified random sample). The representative sample for SHIP-START comprised 7,008 adults, aged 20 to 79 years. The net sample (without migrated or deceased people) consisted of 6,267 eligible individuals, of whom 4,308 individuals participated in the baseline examination between October 1997 and March 2001. The representative sample of SHIP-TREND includes 8,016 adults, aged 20 to 79 years. In total 4,420 individuals participated in the baseline examination between September 2008 and September 2012. There is no overlap among both SHIP cohorts. Both studies complied with the Declaration of Helsinki and were approved by the ethics committee of the University of Greifswald. All participants provided written informed consent.

Lipoprotein subclasses

After thawing, 250 µl of plasma were mixed with 250 µl of phosphate buffer [prepared with D2O and contained sodium 3-trimethylsilyl-(2,2,3,3-D4)-1-propionate (TSP) as reference, (pH 7.4)]. Spectra were recorded on a Bruker AVANCE-II 600 NMR spectrometer operated by TOPSPIN 3.2 software (both Bruker Biospin, Rheinstetten, Germany), equipped with 5-mm z-gradient probe (Bruker Biospin, Rheinstetten, Germany) and automated tuning and matching (ATMA) unit. Specimens were automatically delivered to the spectrometer via SampleJet (Bruker Biospin, Rheinstetten, Germany) into standard 5 mm NMR tubes. The acquisition temperature was set to 310°K. A standard one-dimensional ^{1}H-NMR pulse sequence with suppression of the water peak (NOESYPREAST) was used. The sequence has the form –RD-gz,1-90°-t-90°-tm-gz,2-ACQ, where RD is the relaxation delay (4 s) t is a short delay (~3 µs), 90° represents the 90° RF hard pulse, tm is the mixing time (10 ms), gz,1 and gz,2 are the magnetic field z gradients both applied for 1 ms and ACQ is the acquisition period (2.7 s)

collecting 98304 data points at a sweep width of 30 ppm. The receiver gain is set at 90.5 for all experiments. For pre-processing, a line broadening of 0.3 Hz, a zero filling to produce 128 k data points and a first-order phase correction of 0.0 was applied. Spectral processing included zerofilling, linebroadening, Fourier transform and referencing of the chemical shift and determination of the spectral intensity per 1 mmol protons for quantitative referencing. Chemical shifts of plasma spectra were referenced to the CH3-group signal of alanine adjusting it to 1.48 ppm. Finally, the spectrum is submitted to data analysis for lipoprotein subclass analysis B.I.LISA™ (Bruker BioSpin GmbH, Rheinstetten, Germany). Based on replicates of a pooled plasma sample, which were measured during the year 2023 on two different 600 MHz NMR spectrometer, the coefficients of variation (CV) for total cholesterol, LDL-cholesterol, HDL-cholesterol and triglycerides varied between 2.85%- 4.69%, 3.77% - 6.13%, 3.20% - 5.43% and 4.85% – 5.20%, respectively. The clinically most established lipid parameters (total cholesterol, LDL-cholesterol, HDL-cholesterol and triglycerides) measured by ^{1}H-NMR were compared with standard laboratory measurements (Dimension Vista 1500, Siemens Healthineers, Erlangen, Germany) using Passing-Bablok regression and Pearson correlation based on the whole SHIP-START or SHIP-TREND cohorts. Good agreement was archived for all four parameters (Pearson correlation coefficient r ranged between 0.81 and 0.97). Within the LDL, VLDL and HDL particles strong correlations between the cholesterol and the phospholipid content across all subclasses were found with Pearson correlation coefficients > 0.9 (figure **S8**). Moreover, for VLDL both cholesterol and phospholipid content were strongly correlated to TG content. Therefore, the phospholipid content across all particles and the VLDL subclasses TG content were not considered the present analyses.

References:

1. John U, Greiner B, Hensel E, Ludemann J, Piek M, Sauer S, et al. Study of Health In Pomerania (SHIP): a health examination survey in an east German region: objectives and design. *Soz Praventivmed*. 2001;46(3): 186-194.
2. Völzke H, Alte D, Schmidt CO, Radke D, Lorbeer R, Friedrich N, et al. Cohort Profile: The Study of Health in Pomerania. *Int J Epidemiol*. 2011;40(2): 294-307.

Table S1: Cox regression models for the linear associations of lipoprotein subclasses with all-cause, cardiovascular disease (CVD) and cancer mortality in SHIP-START and SHIP-TREND.

	All-cause mortality				CVD mortality				Cancer mortality			
	SHIP-START		SHIP-TREND		SHIP-START		SHIP-TREND		SHIP-START		SHIP-TREND	
	HR (95% CI) per SD increase	*non-linear confounders*	HR (95% CI) per SD increase	*non-linear confounders*	HR (95% CI) per SD increase	*non-linear confounders*	HR (95% CI) per SD increase	*non-linear confounders*	HR (95% CI) per SD increase	*non-linear confounders*	HR (95% CI) per SD increase	*non-linear confounders*
Total Triglycerides	1.044 (0.975; 1.117)	*alc*	1.018 (0.927; 1.118)	-	1.161 (1.041; 1.296)*	*alc*	1.070 (0.947; 1.209)	-	0.954 (0.838; 1.087)	*age, alc*	1.037 (0.861; 1.250)	*alc*
VLDL Triglycerides	1.031 (0.963; 1.104)	*alc*	1.005 (0.912; 1.107)	-	1.154 (1.035; 1.288)*	*alc*	1.065 (0.931; 1.219)	-	0.937 (0.822; 1.068)	*age, alc*	1.040 (0.864; 1.252)	*alc*
IDL Triglycerides	1.054 (0.986; 1.126)	*age,alc*	1.018 (0.928; 1.118)	-	1.152 (1.033; 1.285)*	*alc*	1.082 (0.960; 1.219)	-	0.982 (0.865; 1.115)	*age, alc*	0.995 (0.814; 1.216)	*alc*
LDL Triglycerides	1.144 (1.069; 1.224)*	*age,alc*	*spline*	-	1.227 (1.089; 1.383)*	*alc*	1.230 (1.028; 1.472)	-	1.086 (0.960; 1.229)	*age, alc*	1.146 (0.939; 1.398)	*alc*
LDL-1 Triglycerides	1.097 (1.023; 1.176)*	*age,alc*	1.138 (1.046; 1.238)*	-	1.160 (1.027; 1.312)	*alc*	1.142 (1.004; 1.299)	-	1.043 (0.920; 1.183)	*age, alc*	1.160 (0.981; 1.372)	*alc*
LDL-2 Triglycerides	1.111 (1.038; 1.190)*	*alc*	1.229 (1.117; 1.353)*	-	1.117 (0.986; 1.266)	*alc*	1.274 (1.065; 1.523)*	-	1.087 (0.963; 1.226)	*age, alc*	1.197 (1.002; 1.431)	*alc*
LDL-3 Triglycerides	1.211 (1.129; 1.300)*	*age,alc*	1.095 (0.986; 1.216)	-	1.205 (1.060; 1.369)*	*alc*	1.119 (0.907; 1.381)	-	1.194 (1.056; 1.351)*	*alc*	1.015 (0.823; 1.253)	*alc*
LDL-4 Triglycerides	1.099 (1.030; 1.173)*	*alc*	spline	-	1.159 (1.031; 1.303)	*alc*	1.114 (0.902; 1.377)	-	1.075 (0.958; 1.207)	*age, alc*	1.122 (0.923; 1.365)	*alc*
LDL-5 Triglycerides	1.128 (1.058; 1.202)*	*age,alc*	1.044 (0.935; 1.166)	-	1.195 (1.068; 1.336)*	*alc*	*spline*	-	1.093 (0.975; 1.226)	*age, alc*	1.085 (0.878; 1.340)	*alc*
LDL-6 Triglycerides	1.163 (1.091; 1.239)*	*age,alc*	*spline*	-	1.248 (1.123; 1.387)*	*alc*	*spline*	-	1.061 (0.942; 1.196)	*age, alc*	0.997 (0.798; 1.246)	*alc*
HDL Triglycerides	1.101 (1.024; 1.184)*	*alc*	1.053 (0.946; 1.172)	-	1.079 (0.940; 1.238)	*alc*	1.030 (0.846; 1.255)	-	1.099 (0.965; 1.251)	*age, alc*	1.130 (0.918; 1.392)	*alc*
HDL-1 Triglycerides	1.119 (1.045; 1.198)*	*alc*	1.126 (1.020; 1.243)	-	1.049 (0.915; 1.203)	*alc*	1.110 (0.932; 1.322)	-	1.102 (0.975; 1.247)	*age, alc*	1.088 (0.879; 1.348)	*alc*
HDL-2 Triglycerides	*spline*	*alc*	1.097 (0.986; 1.221)	-	1.112 (0.969; 1.275)	*alc*	1.071 (0.881; 1.303)	-	1.070 (0.936; 1.222)	*age, alc*	1.160 (0.941; 1.429)	*alc*
HDL-3 Triglycerides	1.060 (0.983; 1.144)	*alc*	1.024 (0.914; 1.147)	-	1.116 (0.973; 1.280)	*alc*	0.974 (0.780; 1.216)	-	1.057 (0.923; 1.209)	*age, alc*	1.175 (0.957; 1.443)	*alc*
HDL-4 Triglycerides	0.998 (0.928; 1.073)	*alc*	0.934 (0.836; 1.043)	-	1.037 (0.912; 1.180)	*alc*	0.927 (0.751; 1.145)	-	1.047 (0.923; 1.186)	*age, alc*	1.108 (0.907; 1.353)	*alc*
Total Cholesterol	1.038 (0.970; 1.110)	*alc*	*spline*	-	1.067 (0.946; 1.204)	*alc*	1.055 (0.847; 1.315)	-	0.973 (0.864; 1.095)	*age, alc*	0.781 (0.624; 0.976)	*alc*
VLDL Cholesterol	1.012 (0.944; 1.085)	*alc*	0.990 (0.891; 1.100)	-	1.166 (1.040; 1.308)*	*alc*	1.091 (0.924; 1.287)	-	0.897 (0.787; 1.021)	*age, alc*	1.068 (0.878; 1.299)	*alc*
VLDL-1 Cholesterol	1.000 (0.933; 1.072)	*alc*	0.990 (0.896; 1.094)	-	1.115 (0.998; 1.245)	*alc*	1.066 (0.932; 1.219)	-	0.899 (0.787; 1.028)	*age, alc*	0.996 (0.816; 1.215)	*alc*
VLDL-2 Cholesterol	1.026 (0.960; 1.097)	*alc*	1.018 (0.917; 1.129)	-	1.167 (1.043; 1.306)*	*alc*	1.104 (0.919; 1.325)	-	0.918 (0.811; 1.040)	*age, alc*	1.073 (0.882; 1.304)	*alc*
VLDL-3 Cholesterol	1.029 (0.962; 1.102)	*alc*	1.008 (0.909; 1.119)	-	1.180 (1.053; 1.323)*	*alc*	1.109 (0.922; 1.333)	-	0.923 (0.815; 1.046)	*age, alc*	1.074 (0.882; 1.308)	*alc*
VLDL-4 Cholesterol	1.033 (0.965; 1.104)	*alc*	0.985 (0.887; 1.094)	-	1.166 (1.041; 1.305)*	*alc*	1.093 (0.892; 1.339)	-	0.936 (0.828; 1.059)	*age, alc*	1.119 (0.927; 1.352)	*alc*
VLDL-5 Cholesterol	1.016 (0.952; 1.085)	*alc*	0.951 (0.859; 1.053)	-	1.028 (0.920; 1.150)	*alc*	0.934 (0.759; 1.149)	-	0.984 (0.874; 1.108)	*age, alc*	1.004 (0.825; 1.221)	*alc*
IDL Cholesterol	1.038 (0.967; 1.114)	*alc*	*spline*	-	1.195 (1.061; 1.346)*	*alc*	1.119 (0.970; 1.291)	-	0.934 (0.820; 1.064)	*age, alc*	1.017 (0.826; 1.253)	*alc*
LDL Cholesterol	1.038 (0.973; 1.108)	*alc*	0.885 (0.798; 0.980)	-	1.061 (0.944; 1.191)	*alc*	0.994 (0.808; 1.222)	-	1.017 (0.907; 1.139)	*age, alc*	0.815 (0.662; 1.002)	*alc*
LDL-1 Cholesterol	0.990 (0.924; 1.061)	*alc*	0.991 (0.883; 1.111)	-	0.960 (0.848; 1.086)	*alc*	1.142 (0.912; 1.431)	-	0.972 (0.860; 1.099)	*age, alc*	0.934 (0.747; 1.167)	*alc*
LDL-2 Cholesterol	0.993 (0.929; 1.062)	*alc*	0.978 (0.877; 1.091)	-	0.969 (0.860; 1.092)	*alc*	1.168 (0.943; 1.447)	-	0.975 (0.866; 1.097)	*age, alc*	0.804 (0.648; 0.997)	-
LDL-3 Cholesterol	1.012 (0.948; 1.079)	*alc*	0.940 (0.839; 1.054)	*wc*	1.001 (0.893; 1.122)	*alc*	1.060 (0.846; 1.328)	-	0.990 (0.884; 1.109)	*age, alc*	0.766 (0.607; 0.965)	*alc*
LDL-4 Cholesterol	1.029 (0.965; 1.098)	*alc*	0.884 (0.792; 0.986)	-	1.034 (0.923; 1.160)	*alc*	0.951 (0.761; 1.189)	-	1.040 (0.929; 1.165)	*age, alc*	0.814 (0.655; 1.012)	*alc*
LDL-5 Cholesterol	1.056 (0.990; 1.126)	*alc*	0.865 (0.780; 0.959)*	-	1.109 (0.990; 1.242)	*alc*	*spline*	-	1.064 (0.949; 1.193)	*age, alc*	0.872 (0.709; 1.071)	*alc*
LDL-6 Cholesterol	1.102 (1.034; 1.175)*	*age,alc*	*spline*	-	1.151 (1.033; 1.283)*	*alc*	*spline*	-	1.018 (0.907; 1.143)	*age, alc*	0.915 (0.737; 1.135)	*alc*
HDL Cholesterol	*spline*	*alc*	0.813 (0.711; 0.930)*	-	*spline*	*alc*	0.926 (0.716; 1.197)	-	1.053 (0.923; 1.200)	*age, alc*	0.661 (0.509; 0.857)*	*alc*
HDL-1 Cholesterol	1.084 (1.008; 1.166)	*alc*	1.069 (0.952; 1.201)	-	0.901 (0.779; 1.041)	*alc*	1.237 (1.010; 1.514)	-	1.070 (0.941; 1.217)	*age, alc*	0.780 (0.603; 1.010)	*alc*
HDL-2 Cholesterol	1.046 (0.969; 1.128)	*alc*	0.895 (0.785; 1.020)	-	0.898 (0.780; 1.034)	*alc*	1.001 (0.777; 1.290)	-	1.042 (0.911; 1.191)	*age, alc*	0.736 (0.571; 0.949)	*alc*
HDL-3 Cholesterol	0.984 (0.913; 1.061)	*alc*	0.805 (0.707; 0.917)*	-	0.882 (0.767; 1.014)	*alc*	0.807 (0.621; 1.048)	-	1.028 (0.902; 1.170)	*age, alc*	0.713 (0.555; 0.918)*	*alc*
HDL-4 Cholesterol	0.904 (0.846; 0.966)*	*alc*	0.728 (0.657; 0.806)*	-	0.819 (0.730; 0.919)*	*alc*	0.712 (0.579; 0.876)*	-	0.980 (0.871; 1.103)	*age, alc*	0.743 (0.607; 0.908)*	*alc*

Hazard ratio (HR) with 95% confidence interval (CI) for one standard deviation increase (SD) in lipoprotein subclass. *spline*: spline function significant (Table S2). Models were adjusted for age, sex, waist circumference, smoking, physical activity, alcohol consumption, diabetes mellitus, renal diseases, liver diseases, cardiovascular disease and systolic blood pressure. When appropriate, spline terms for age, waist circumference and alcohol consumption were included (see column “non-linear confounder”). VLDL = very low density lipoprotein, IDL = intermediate density lipoprotein, LDL = low density lipoprotein, HDL = high density lipoprotein, PL = Phospholipids. * FDR < 0.05.

Continue table S1:

	All-cause mortality				CVD mortality				Cancer mortality			
	SHIP-START		SHIP-TREND		SHIP-START		SHIP-TREND		SHIP-START		SHIP-TREND	
	HR (95% CI) per SD increase	*non-linear confounders*	HR (95% CI) per SD increase	*non-linear confounders*	HR (95% CI) per SD increase	*non-linear confounders*	HR (95% CI) per SD increase	*non-linear confounders*	HR (95% CI) per SD increase	*non-linear confounders*	HR (95% CI) per SD increase	*non-linear confounders*
Total Apo-B	1.094 (1.020; 1.174)	*age,alc*	*spline*	-	1.178 (1.042; 1.333)*	*alc*	*spline*	-	1.013 (0.895; 1.146)	*age, alc*	0.912 (0.737; 1.129)	*alc*
VLDL Apo-B	1.036 (0.966; 1.110)	*alc*	1.008 (0.909; 1.118)	-	1.187 (1.059; 1.330)*	*alc*	1.076 (0.910; 1.271)	-	0.927 (0.813; 1.056)	*age, alc*	1.120 (0.930; 1.349)	*alc*
IDL Apo-B	1.061 (0.988; 1.139)	*alc*	*spline*	-	1.197 (1.063; 1.349)*	*alc*	*spline*	-	0.977 (0.857; 1.112)	*age, alc*	1.087 (0.888; 1.332)	*alc*
LDL Apo-B	1.099 (1.028; 1.175)*	*age,alc*	0.938 (0.848; 1.039)	-	1.134 (1.009; 1.276)	*alc*	1.054 (0.861; 1.292)	-	1.045 (0.930; 1.175)	*age, alc*	0.875 (0.713; 1.074)	*alc*
LDL-1 Apo-B	1.018 (0.950; 1.091)	*alc*	1.084 (0.971; 1.212)	-	0.997 (0.880; 1.130)	*alc*	1.246 (1.007; 1.543)	-	0.973 (0.860; 1.102)	*age, alc*	1.030 (0.832; 1.275)	*alc*
LDL-2 Apo-B	1.007 (0.943; 1.077)	*alc*	0.998 (0.896; 1.111)	-	0.997 (0.886; 1.122)	*alc*	1.165 (0.942; 1.441)	-	0.996 (0.886; 1.119)	*age, alc*	0.872 (0.707; 1.077)	*alc*
LDL-3 Apo-B	1.018 (0.954; 1.086)	*alc*	0.972 (0.870; 1.086)	-	1.022 (0.911; 1.147)	*alc*	1.096 (0.882; 1.363)	-	0.988 (0.882; 1.107)	*age, alc*	0.802 (0.644; 0.997)	*alc*
LDL-4 Apo-B	1.035 (0.969; 1.105)	*alc*	0.898 (0.805; 1.002)	-	1.058 (0.941; 1.191)	*alc*	0.957 (0.764; 1.197)	-	1.045 (0.931; 1.173)	*age, alc*	0.848 (0.684; 1.053)	*alc*
LDL-5 Apo-B	1.083 (1.014; 1.156)	*age,alc*	0.878 (0.790; 0.977)	-	1.143 (1.020; 1.282)	*alc*	spline	-	1.070 (0.953; 1.202)	*age, alc*	0.902 (0.731; 1.112)	*alc*
LDL-6 Apo-B	1.109 (1.040; 1.182)*	*age,alc*	*spline*	-	1.176 (1.056; 1.308)*	*alc*	*spline*	-	1.026 (0.912; 1.154)	*age, alc*	0.922 (0.741; 1.148)	*alc*
Total Apo-A1	*spline*	*alc*	0.736 (0.641; 0.844)*	-	0.768 (0.664; 0.889)*	*alc*	*spline*	-	1.104 (0.965; 1.264)	*age, alc*	0.681 (0.525; 0.885)*	*alc*
HDL-1 Apo-A1	1.099 (1.019; 1.186)	*alc*	1.032 (0.916; 1.162)	-	0.890 (0.764; 1.037)	*alc*	*spline*	-	1.097 (0.961; 1.252)	*age, alc*	0.833 (0.645; 1.077)	*alc*
HDL-2 Apo-A1	1.108 (1.024; 1.198)*	*alc*	0.946 (0.825; 1.084)	-	0.911 (0.784; 1.058)	*alc*	0.936 (0.717; 1.222)	-	1.180 (1.031; 1.349)	*age, alc*	0.743 (0.566; 0.974)	*alc*
HDL-3 Apo-A1	1.008 (0.932; 1.090)	*alc*	0.835 (0.733; 0.952)*	-	0.901 (0.778; 1.042)	*alc*	0.780 (0.597; 1.021)	-	1.056 (0.923; 1.209)	*age, alc*	0.766 (0.591; 0.994)	*alc*
HDL-4 Apo-A1	0.911 (0.853; 0.972)*	*alc*	0.710 (0.640; 0.788)*	-	0.809 (0.721; 0.908)*	*alc*	0.673 (0.546; 0.829)*	-	1.013 (0.900; 1.140)	*age, alc*	0.778 (0.635; 0.954)	*alc*
Total Apo-A2	*spline*	*alc*	0.742 (0.652; 0.843)*	-	0.908 (0.788; 1.047)	*alc*	0.755 (0.582; 0.981)	-	1.010 (0.887; 1.150)	*age, alc*	0.705 (0.550; 0.903)*	*alc*
HDL-1 Apo-A2	1.143 (1.063; 1.228)*	*alc*	1.099 (0.979; 1.234)	-	1.037 (0.902; 1.191)	*alc*	1.160 (0.938; 1.434)	-	1.112 (0.979; 1.262)	*age, alc*	0.826 (0.636; 1.072)	*alc*
HDL-2 Apo-A2	1.094 (1.019; 1.174)	*alc*	1.047 (0.918; 1.193)	-	1.076 (0.944; 1.227)	*alc*	1.077 (0.826; 1.404)	-	1.072 (0.945; 1.216)	*age, alc*	0.849 (0.654; 1.103)	*alc*
HDL-3 Apo-A2	1.014 (0.940; 1.095)	*alc*	0.852 (0.744; 0.975)	-	1.045 (0.909; 1.203)	*alc*	0.851 (0.644; 1.124)	-	1.009 (0.884; 1.151)	*age, alc*	0.808 (0.618; 1.056)	*alc*
HDL-4 Apo-A2	0.882 (0.822; 0.947)*	*alc*	0.733 (0.658; 0.815)*	-	0.845 (0.744; 0.961)*	*alc*	0.707 (0.567; 0.882)*	-	0.971 (0.857; 1.100)	*age, alc*	0.778 (0.633; 0.957)	*alc*
Phospholipids												
VLDL Phospholipids	1.028 (0.960; 1.100)	*alc*	0.993 (0.897; 1.100)	-	1.159 (1.037; 1.295)*	*alc*	1.064 (0.898; 1.260)	-	0.924 (0.812; 1.051)	*age, alc*	1.075 (0.890; 1.300)	*alc*
IDL Phospholipids	1.037 (0.969; 1.110)	*alc*	0.962 (0.865; 1.071)	-	1.169 (1.043; 1.310)*	*alc*	1.094 (0.947; 1.264)	-	0.937 (0.827; 1.062)	*age, alc*	0.953 (0.769; 1.183)	*alc*
LDL Phospholipids	1.055 (0.990; 1.126)	*alc*	0.912 (0.824; 1.011)	-	1.060 (0.945; 1.188)	*alc*	1.030 (0.841; 1.261)	-	1.042 (0.931; 1.167)	*age, alc*	0.830 (0.676; 1.019)	*alc*
HDL Phospholipids	1.071 (0.991; 1.158)	*alc*	0.889 (0.774; 1.021)	-	0.850 (0.735; 0.983)	*alc*	0.934 (0.712; 1.225)	-	1.134 (0.990; 1.299)	*age, alc*	0.693 (0.525; 0.913)*	*alc*

Hazard ratio (HR) with 95% confidence interval (CI) for one standard deviation increase (SD) in lipoprotein subclass. *spline*: spline function significant (Table S2). Models were adjusted for age, sex, waist circumference, smoking, physical activity, alcohol consumption, diabetes mellitus, renal diseases, liver diseases, CVD and systolic blood pressure. When appropriate, spline terms for age, waist circumference and alcohol consumption were included (see column "non-linear confounder"). VLDL = very low density lipoprotein, IDL = intermediate density lipoprotein, LDL = low density lipoprotein, HDL = high density lipoprotein, PL = Phospholipids. * FDR < 0.05.

Table S2: Cox regression models for the association of lipoprotein subclasses with all-cause and CVD mortality using restricted cubic splines (see methods) in SHIP-START and SHIP-TREND.

		SHIP-START					
		**All-cause mortality**			**CVD mortality**		
		**LRT p**	**HR (95% CI)**	**global p#**	**LRT p**	**HR (95% CI)**	**global p#**
HDL-2 Triglycerides	linear term	0.006	1.443 (1.165; 1.787)*	0.002		*no spline term*	
	spline term		0.958 (0.928; 0.989)*				
HDL Cholesterol	linear term	0.005	0.818 (0.697; 0.960)*	0.014	0.027	0.609 (0.463; 0.800)*	<0.001
	spline term		1.033 (1.011; 1.056)*			1.049 (1.007; 1.093)*	
Total Apo-A1	linear term	0.002	0.807 (0.692; 0.941)*	0.005		*no spline term*	
	spline term		1.034 (1.013; 1.055)*				
Total Apo-A2	linear term	0.014	0.835 (0.729; 0.957)*	0.028		*no spline term*	
	spline term		1.023 (1.005; 1.042)*				

		SHIP-TREND					
		**All-cause mortality**			**CVD mortality**		
		**LRT p**	**HR (95% CI)**	**global p#**	**LRT p**	**HR (95% CI)**	**global p#**
LDL Triglycerides	linear term	0.001	0.704 (0.524; 0.945)*	<0.001		*no spline term*	
	spline term		1.068 (1.030; 1.108)*				
LDL-4 Triglycerides	linear term	0.011	0.804 (0.629; 1.028)	0.004		*no spline term*	
	spline term		1.042 (1.011; 1.075)*				
LDL-5 Triglycerides	linear term		*no spline term*		0.009	0.513 (0.295; 0.894)*	0.007
	spline term					1.107 (1.033; 1.187)*	
LDL-6 Triglycerides	linear term	0.004	0.686 (0.492; 0.957)*	<0.001	0.020	0.578 (0.311; 1.075)	<0.001
	spline term		1.081 (1.028; 1.137)*			1.118 (1.023; 1.221)*	
Total Cholesterol	linear term	0.002	0.646 (0.526; 0.794)*	<0.001		*no spline term*	
	spline term		1.044 (1.017; 1.072)*				
IDL Cholesterol	linear term	0.005	0.708 (0.545; 0.919)*	0.009		*no spline term*	
	spline term		1.052 (1.017; 1.087)*				
LDL-5 Cholesterol	linear term		*no spline term*		0.003	0.514 (0.340; 0.777)*	0.004
	spline term					1.086 (1.034; 1.140)*	
LDL-6 Cholesterol	linear term	0.004	0.650 (0.497; 0.850)*	0.007	<0.001	0.405 (0.255; 0.643)*	<0.001
	spline term		1.060 (1.020; 1.100)*			1.149 (1.082; 1.221)*	
Total Apo-B	linear term	0.002	0.659 (0.522; 0.830)*	0.002	0.009	0.610 (0.392; 0.948)*	0.011
	spline term		1.046 (1.018; 1.075)*			1.071 (1.021; 1.122)*	
IDL Apo-B	linear term	0.001	0.672 (0.517; 0.874)*	0.001	0.019	0.622 (0.379; 1.020)	0.011
	spline term		1.058 (1.025; 1.092)*			1.069 (1.014; 1.128)*	
LDL-5 Apo-B	linear term		*no spline term*		0.001	0.471 (0.309; 0.717)*	0.001
	spline term					1.098 (1.044; 1.155)*	
LDL-6 Apo-B	linear term	0.003	0.632 (0.476; 0.839)*	0.006	<0.001	0.410 (0.250; 0.674)*	<0.001
	spline term		1.070 (1.026; 1.116)*			1.156 (1.081; 1.237)*	
Total Apo-A1	linear term		*no spline term*		0.003	0.418 (0.267; 0.656)*	0.001
	spline term					1.115 (1.044; 1.191)*	
HDL-1 Apo-A1	linear term		*no spline term*		0.005	0.453 (0.238; 0.859)*	0.006
	spline term					1.175 (1.054; 1.311)*	

* $p < 0.05$. # Wald test for the joint linear hypotheses of the linear and spline term. Hazard ratio (HR) with 95% confidence interval (CI) for one standard deviation increase in lipoprotein subclass. Models were adjusted for age, sex, waist circumference, smoking, physical activity, alcohol consumption, diabetes mellitus, renal diseases, liver diseases, CVD and systolic blood pressure. For selected confounders non-linear spline terms were included (see table S1). CVD = cardiovascular disease, LRT = likelihood ratio test, IDL = intermediate density lipoprotein, LDL = low density lipoprotein, HDL = high density lipoprotein.

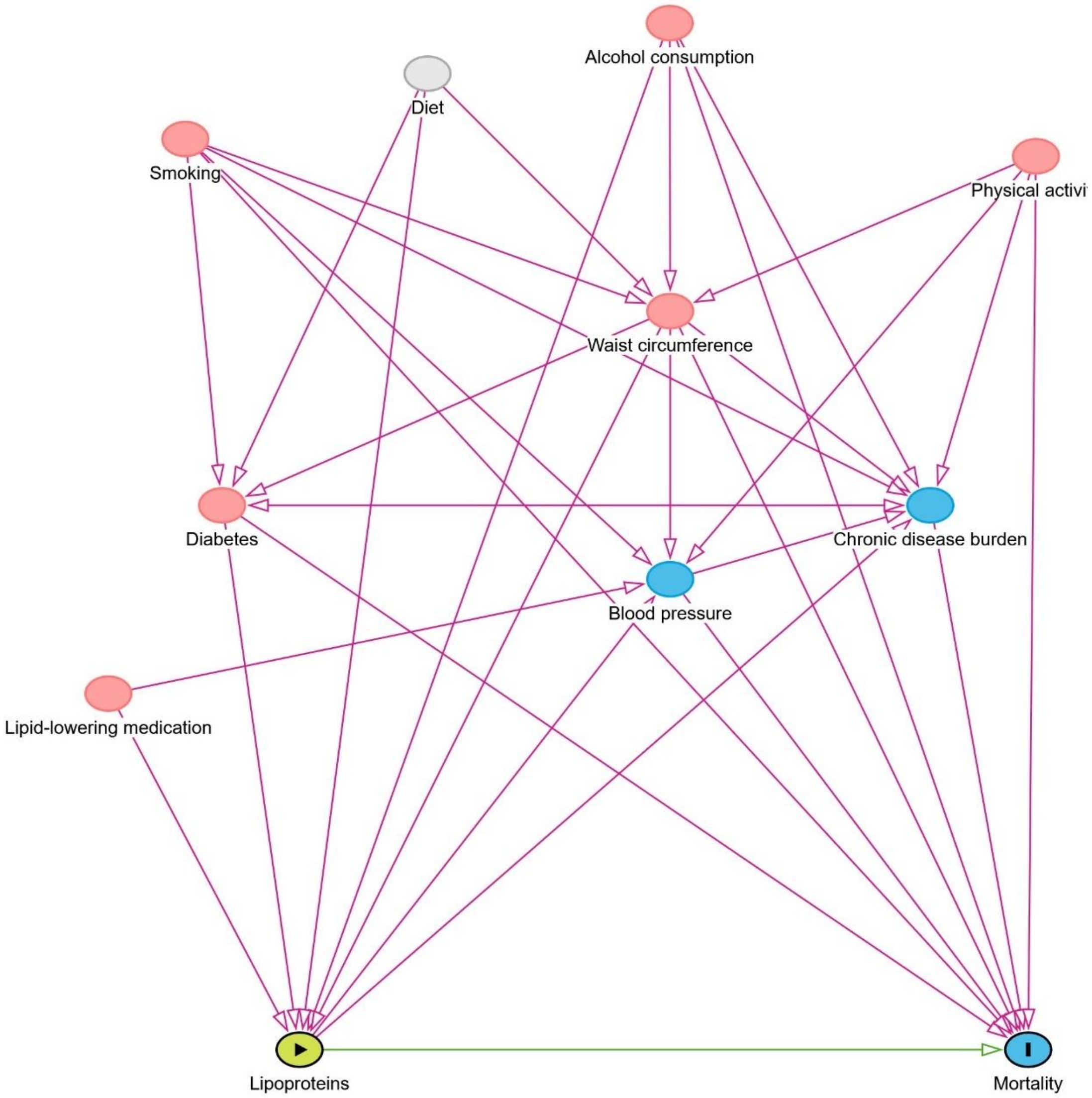

Figure S1: Directed acyclic graph (DAG) showing causal assumptions on the effect of lipoproteins (exposure) on mortality (outcome). The DAG was built using DAGitty v3.0 software (available at http://dagitty.net/development/dags). The selection of the variables was based on biological mechanisms or evidence from previously published data. Age and sex were not displayed for simplicity. The minimal sufficient adjustment set for estimating the *direct effect* (taking into account confounders and mediators) of lipoproteins on mortality included age, sex, alcohol consumption, blood pressure, physical activity, smoking, waist circumference, chronic disease burden and diabetes. Chronic disease burden was defined as combination of cardiovascular, liver and renal diseases.

Green circle with black outline = exposure; blue circle with black outline = outcome; blue circle = mediator; red circle = confounder; green circle = ancestor of exposure; grey circle = unobserved variable.

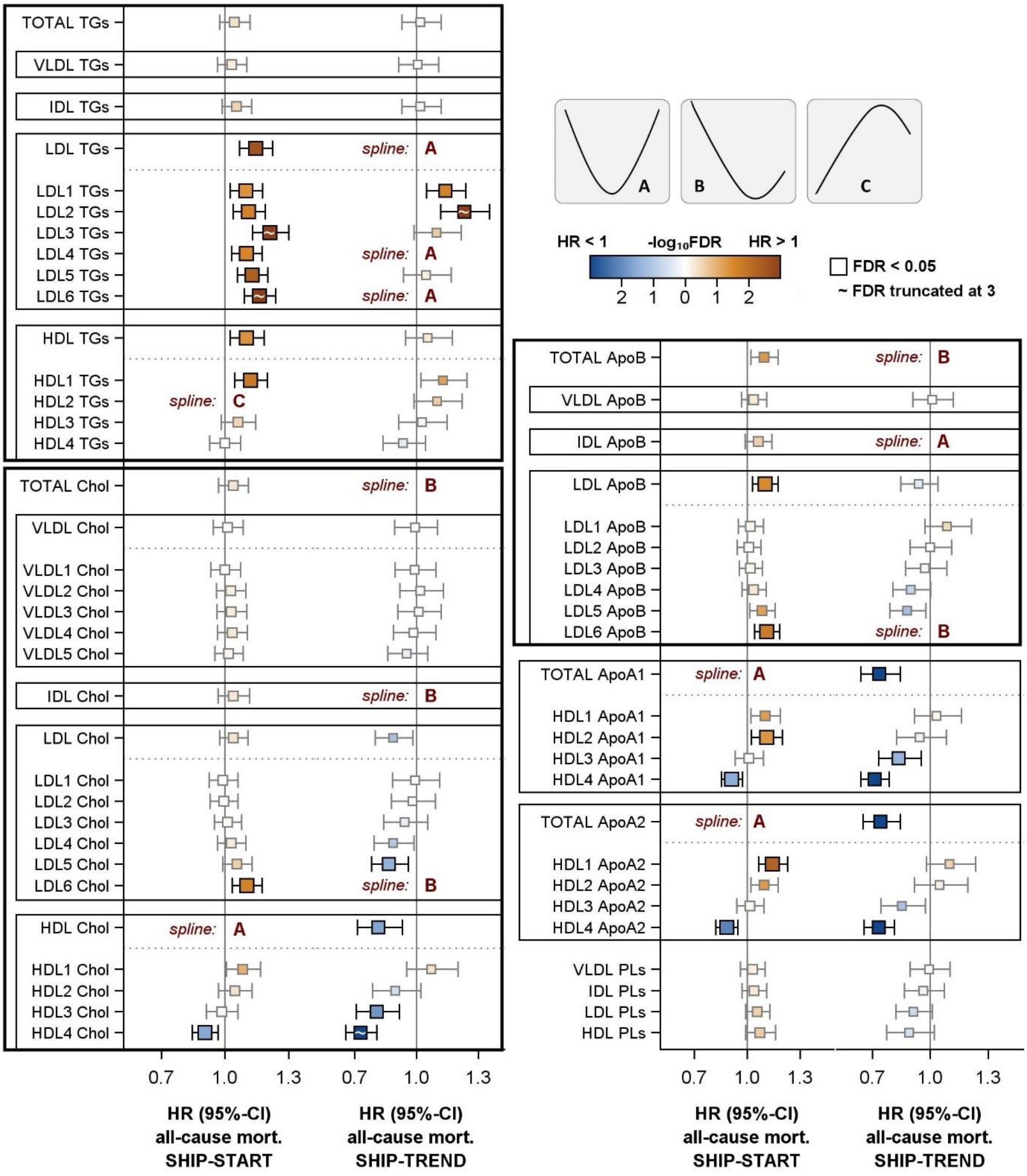

Figure S2. Cox regression models for the associations of lipoprotein subclasses with all-cause mortality in SHIP-START and SHIP-TREND. Hazard ratios (HR) with 95% confidence intervals (CI) for one standard deviation increase in lipoprotein subclasses are displayed. Models were adjusted for age, sex, waist circumference, smoking, physical activity, alcohol consumption, diabetes mellitus, renal diseases, liver diseases, cardiovascular disease and systolic blood pressure. In selected models spline terms for age, waist circumference and alcohol consumption were included. Models with a spline term for lipoproteins (see method section) are marked by spline and function form (see upper right part of the figure). TGs = triglycerides, Chol = cholesterol, VLDL = very low density lipoprotein, IDL = intermediate density lipoprotein, LDL = low density lipoprotein, HDL = high density lipoprotein, PL = Phospholipids.

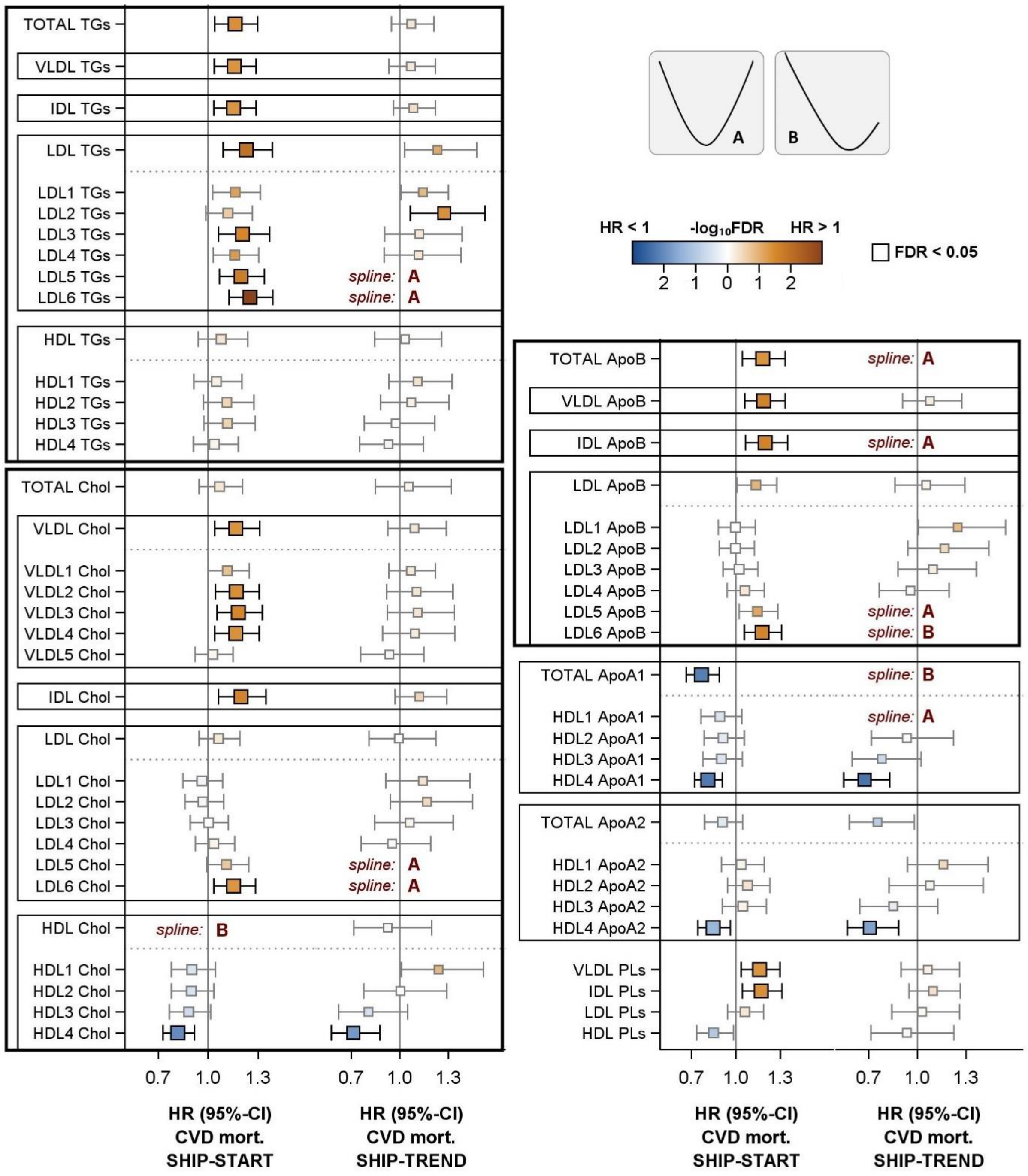

Figure S3. Cox regression models for the associations of lipoprotein subclasses with cardiovascular disease (CVD) mortality in SHIP-START and SHIP-TREND. Hazard ratios (HR) with 95% confidence intervals (CI) for one standard deviation increase in lipoprotein subclasses are displayed. Models were adjusted for age, sex, waist circumference, smoking, physical activity, alcohol consumption, diabetes mellitus, renal diseases, liver diseases, cardiovascular disease and systolic blood pressure. In selected models spline terms for age, waist circumference and alcohol consumption were included. Models with a spline term for lipoproteins (see method section) are marked by spline and function form (see upper right part of the figure). TGs = triglycerides, Chol = cholesterol, VLDL = very low density lipoprotein, IDL = intermediate density lipoprotein, LDL = low density lipoprotein, HDL = high density lipoprotein, PL = Phospholipids.

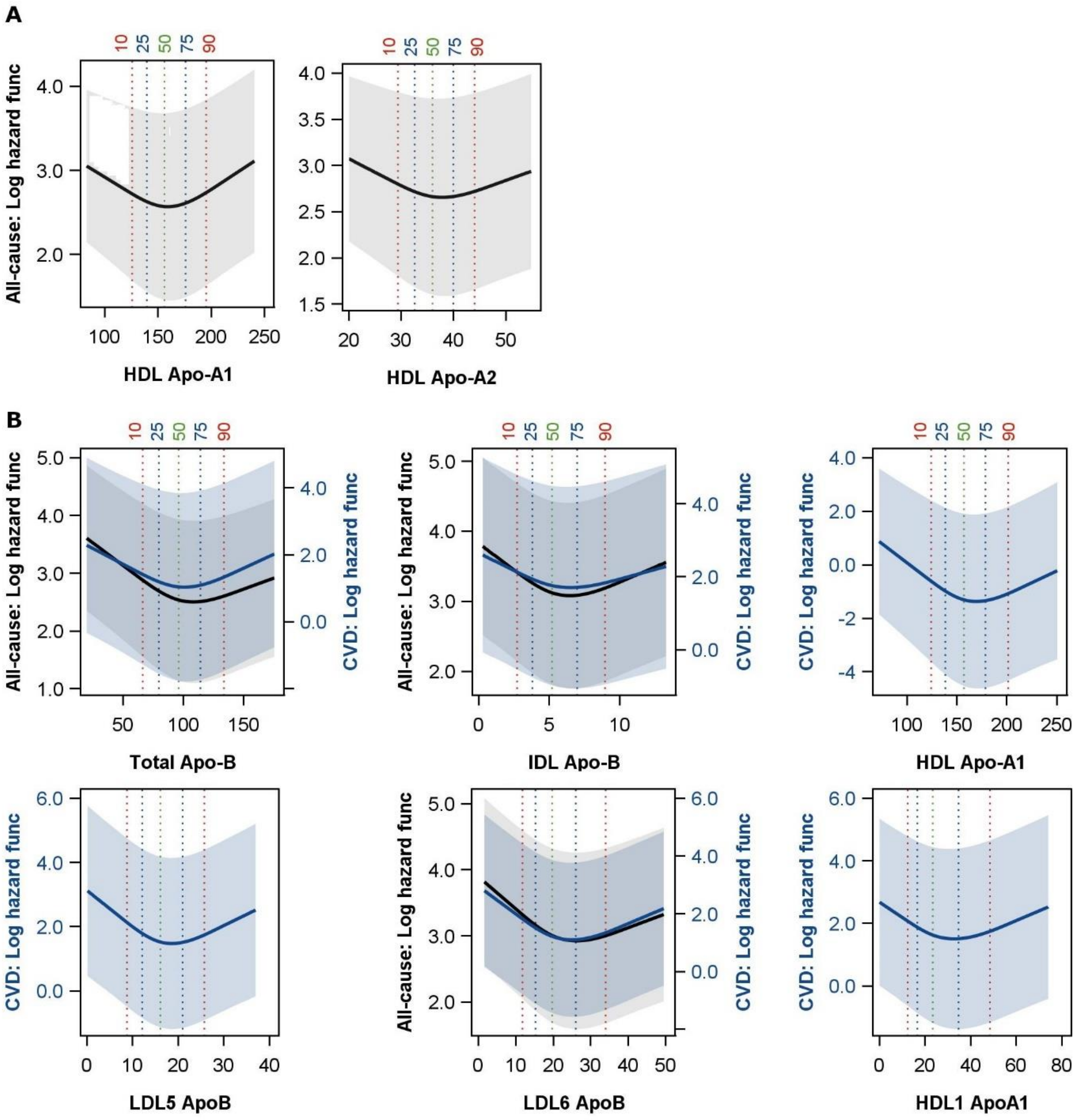

Figure S4. Predicted log hazard function (func) for all-cause (black) and/or cardiovascular disease (CVD) mortality (blue) with 95% confidence interval (shaded area) as a function of lipoprotein subclasses in SHIP-START (A) and SHIP-TREND (B) for selected non-linear associations (see Figure 1 and 2). 10%, 25%, median, 75% and 90% percentile are displayed. The displayed range of the lipoprotein subclasses was restricted to the mean ±3 standard deviations for simplification. Cox regression models were adjusted for age, sex, waist circumference, smoking, physical activity, alcohol consumption, diabetes mellitus, renal diseases, liver diseases, cardiovascular disease and systolic blood pressure. In selected models, a spline term for alcohol consumption was included. LDL = low density lipoprotein, HDL = high density lipoprotein.

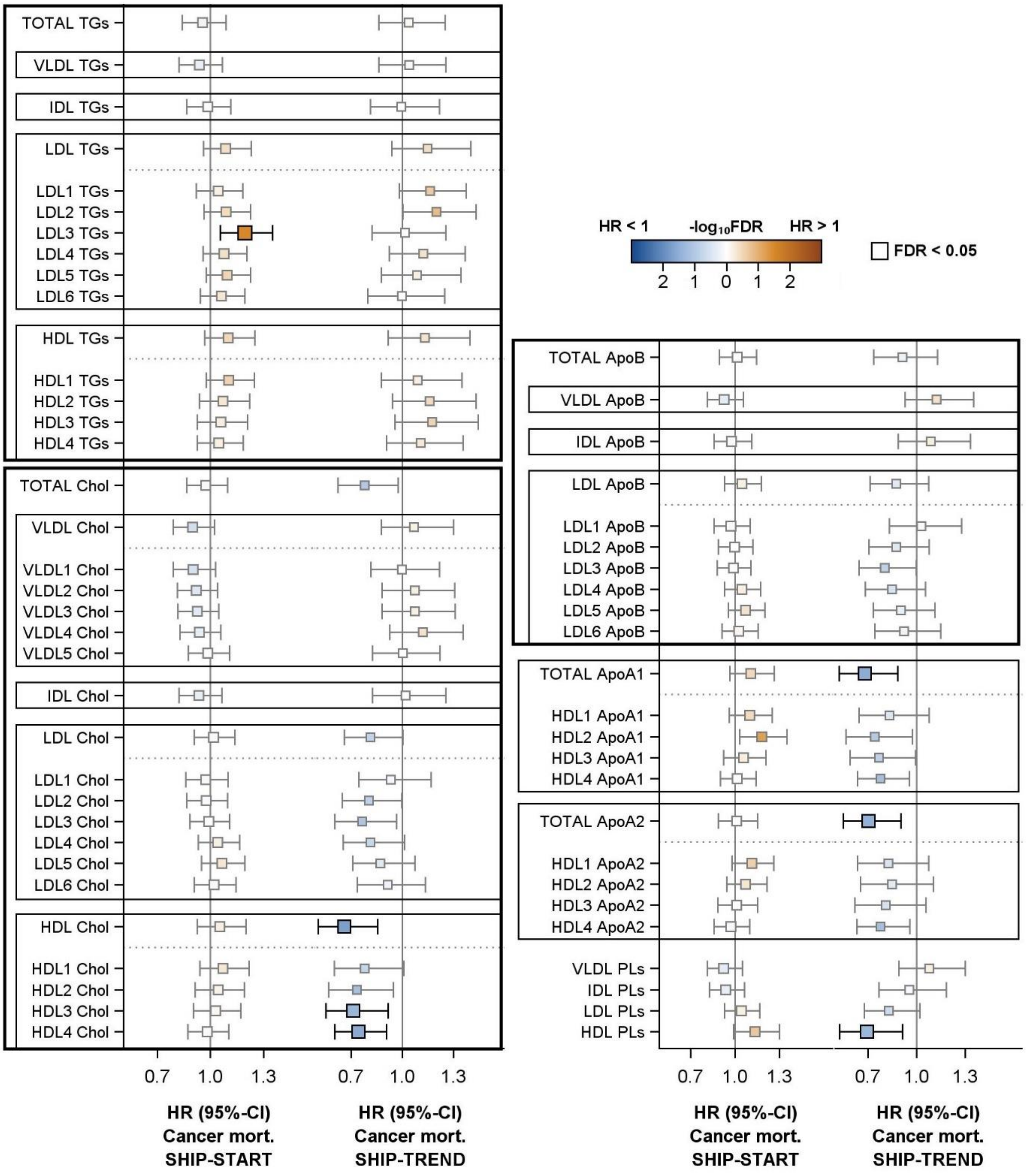

Figure S5. Cox regression models for the associations of lipoprotein subclasses with cancer mortality in SHIP-START and SHIP-TREND. Hazard ratios (HR) with 95% confidence intervals (CI) for one standard deviation increase in lipoprotein subclasses are displayed. Models were adjusted for age, sex, waist circumference, smoking, physical activity, alcohol consumption, diabetes mellitus, renal diseases, liver diseases, cardiovascular disease and systolic blood pressure. In selected models spline terms for age, waist circumference and alcohol consumption were included. TGs = triglycerides, Chol = cholesterol, VLDL = very low density lipoprotein, IDL = intermediate density lipoprotein, LDL = low density lipoprotein, HDL = high density lipoprotein, PL = Phospholipids.

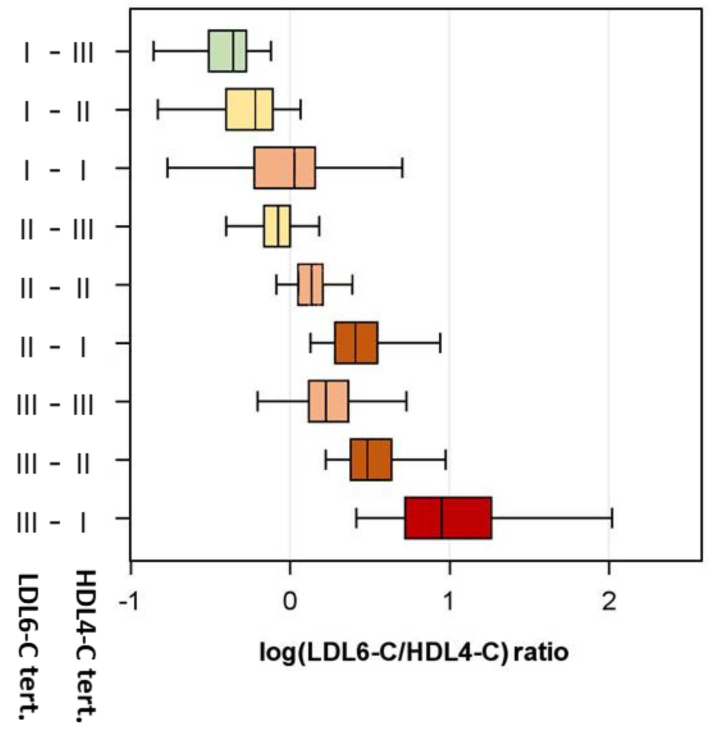

Figure S6. Distribution of the log(HDL4-C/LDL6-C) ratio by tertiles of HDL4 cholesterol and LDL6 cholesterol in SHIP-START. LDL = low density lipoprotein, HDL = high density lipoprotein.

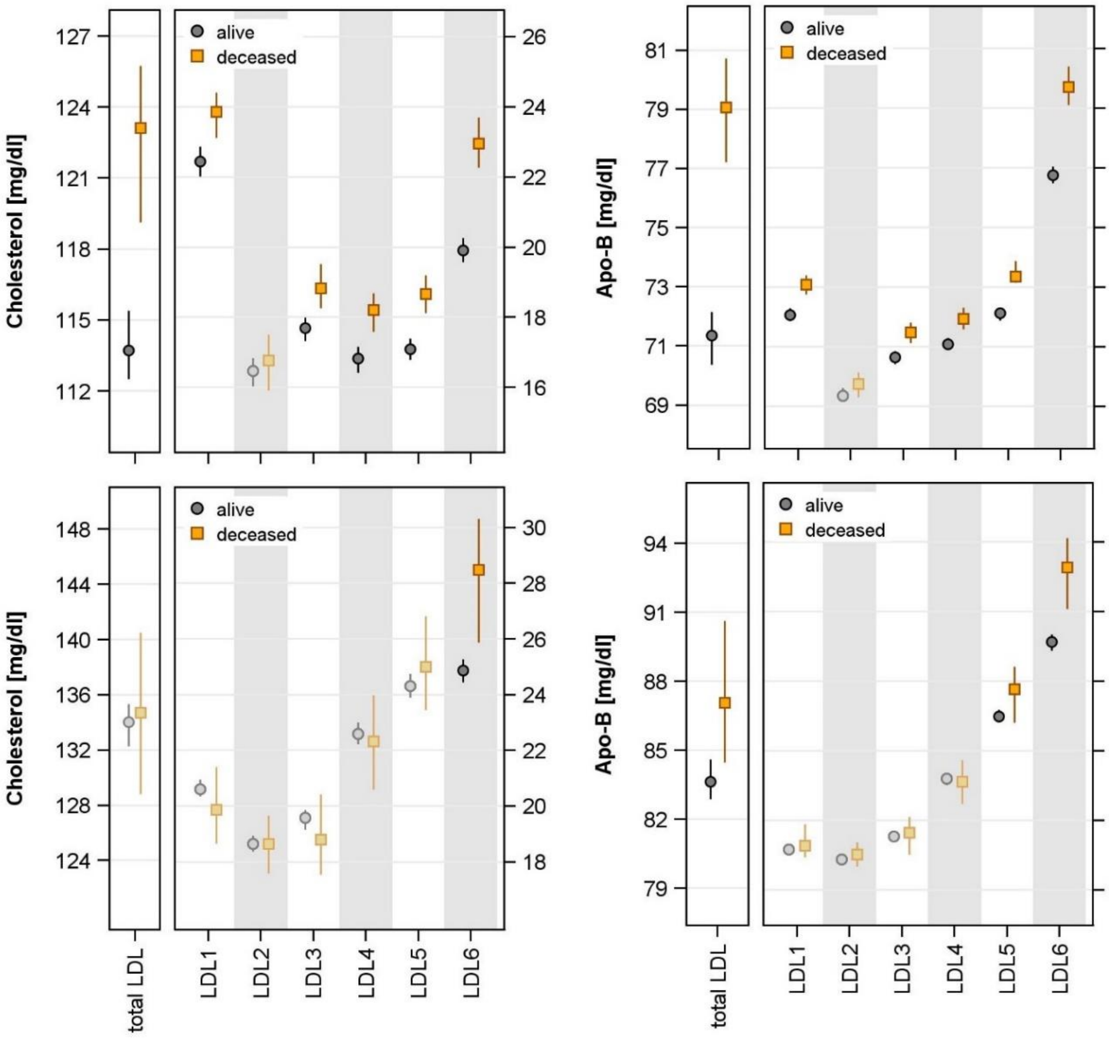

Figure S7. Median levels with 95% confidence interval of the apolipoprotein B (right side) and cholesterol (left side) content across LDL subclasses in subjects without taking lipid-lowering medication by survival status. Levels were separately displayed for SHIP-START (upper row) and SHIP-TREND (lower row).

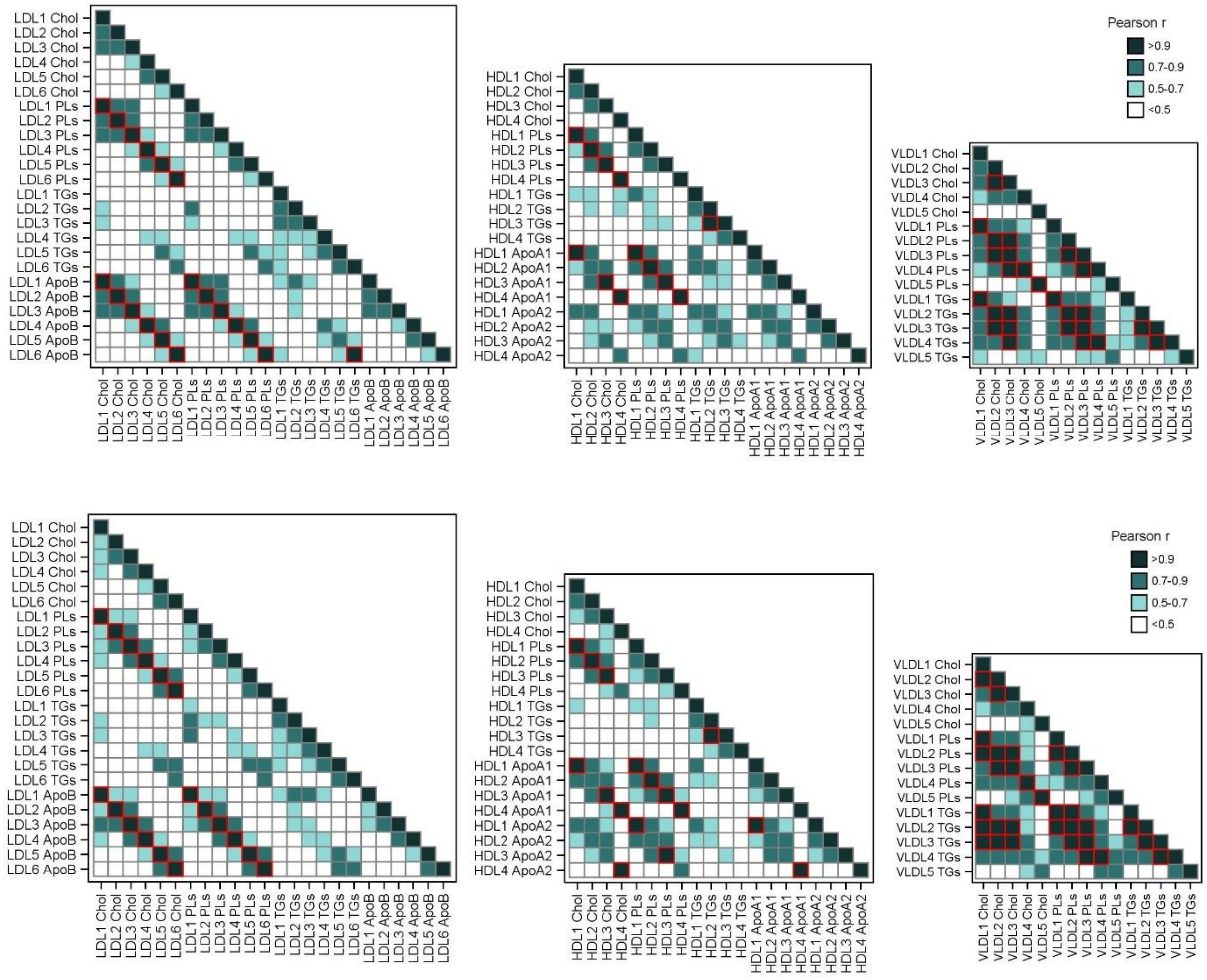

Figure S8. Correlation matrices showing within-particle Pearson correlations in LDL subclasses (left), HDL subclasses (middle) and VLDL subclasses (right).

Upper row – SHIP-START; lower row – SHIP-TREND.
